# Nuclear localization of mitochondrial TCA cycle enzymes modulates pluripotency via histone acetylation

**DOI:** 10.1038/s41467-022-35199-0

**Published:** 2022-12-02

**Authors:** Wei Li, Qi Long, Hao Wu, Yanshuang Zhou, Lifan Duan, Hao Yuan, Yingzhe Ding, Yile Huang, Yi Wu, Jinyu Huang, Delong Liu, Baodan Chen, Jian Zhang, Juntao Qi, Shiwei Du, Linpeng Li, Yang Liu, Zifeng Ruan, Zihuang Liu, Zichao Liu, Yifan Zhao, Jianghuan Lu, Junwei Wang, Wai-Yee Chan, Xingguo Liu

**Affiliations:** 1 GMU-GIBH Joint School of Life Sciences, CAS Key Laboratory of Regenerative Biology, Guangzhou Institutes of Biomedicine and Health, Chinese Academy of Sciences; Guangzhou Medical University, Guangzhou, 510530 China; 2 Guangdong Provincial Key Laboratory of Stem Cell and Regenerative Medicine, China-New Zealand Joint Laboratory on Biomedicine and Health, CUHK-GIBH Joint Research Laboratory on Stem Cells and Regenerative Medicine, Institute for Stem Cell and Regeneration, Guangzhou Institutes of Biomedicine and Health, Chinese Academy of Sciences, Guangzhou, 510530 China; 3 University of Chinese Academy of Sciences, Beijing, 100049 China; 4 Centre for Regenerative Medicine and Health, Hong Kong Institute of Science & Innovation, Chinese Academy of Sciences, Hong Kong SAR, China; 5 CUHK-GIBH Joint Research Laboratory on Stem Cells and Regenerative Medicine, Key Laboratory for Regenerative Medicine, Ministry of Education, School of Biomedical Sciences, Faculty of Medicine, The Chinese University of Hong Kong, Hong Kong SAR, China

**Keywords:** Stem cells, Mitochondria

## Abstract

Pluripotent stem cells hold great promise in regenerative medicine and developmental biology studies. Mitochondrial metabolites, including tricarboxylic acid (TCA) cycle intermediates, have been reported to play critical roles in pluripotency. Here we show that TCA cycle enzymes including Pdha1, Pcb, Aco2, Cs, Idh3a, Ogdh, Sdha and Mdh2 are translocated to the nucleus during somatic cell reprogramming, primed-to-naive transition and totipotency acquisition. The nuclear-localized TCA cycle enzymes Pdha1, Pcb, Aco2, Cs, Idh3a promote somatic cell reprogramming and primed-to-naive transition. In addition, nuclear-localized TCA cycle enzymes, particularly nuclear-targeted Pdha1, facilitate the 2-cell program in pluripotent stem cells. Mechanistically, nuclear Pdha1 increases the acetyl-CoA and metabolite pool in the nucleus, leading to chromatin remodeling at pluripotency genes by enhancing histone H3 acetylation. Our results reveal an important role of mitochondrial TCA cycle enzymes in the epigenetic regulation of pluripotency that constitutes a mitochondria-to-nucleus retrograde signaling mode in different states of pluripotent acquisition.

## Abstract (web summary)

Cellular metabolism is important in pluripotency and cell fate regulation. Here, authors observe chromatin remodeling followed by TCA enzyme translocation from the mitochondria to the nucleus, demonstrating pluripotency regulation by mitochondria to nucleus retrograde signaling.

## Introduction

Pluripotent stem cells (PSCs), including embryonic stem cells (ESCs) and induced pluripotent stem cells (iPSCs), hold great potential in regenerative medicine and developmental biology studies for their unique abilities of self-renewal and the capacity to differentiate into three germ layers and germ cells^1^. Moreover, two-cell (2C) state of embryos, totipotent cells, could differentiate into all cell types of embryonic and extraembryonic tissues^2^. iPSCs, particularly, greatly expand the potential of regenerative medicine by avoiding the potential for immune rejection and the issues of ethics associated with ESCs^3^. Open chromatin structure is associated with plasticity in different states of PSCs, and together with genetic programs and epigenetic modifications, coordinately regulates self-renewal and differentiation^1,4^.

Pluripotency could be achieved by the fusion of the nucleus of a somatic cell with the cytoplasm of an oocyte or PSCs, indicating that the extra-nuclear state of the cell is also a key to pluripotency^5^. Mitochondria are known to play important roles in the metabolic switch required for pluripotency acquisition and primed-to-naive transition^6,7^. Somatic cells mainly rely on oxidative phosphorylation (OXPHOS) in mitochondria to obtain energy while PSCs largely depend on glycolysis in the cytoplasm to produce energy^8,9^. The metabolic switch from OXPHOS to glycolysis is critical for pluripotency acquisition not only through metabolism per se, but also by contributing to the mitochondria-to-nucleus retrograde signaling that regulates epigenetic modifications and gene expression^10–12^. Mitochondrial permeability transition pore (mPTP) opening, a key regulator of mitochondrial homeostasis, can modulate pluripotency acquisition via histone lysine methylation^13^. Mitochondria-derived reactive oxygen species (mito-ROS) and ‘mitoflashes’ also regulate pluripotency acquisition by influencing epigenetic modifications, the cell cycle, and nuclear DNA damage^13,14^. Mitochondria also provide important metabolites for epigenetic modifications in the nucleus in pluripotency acquisition and embryonic development, such as α-ketoglutarate (α-KG), S-adenosyl methionine (SAM), acetyl-CoA, malic acid, and Nicotinamide adenine dinucleotide (NAD)+^15–18^. α-KG, generated by isocitrate dehydrogenase 3 (Idh3), is an important substrate for DNA and histone demethylation. SAM, derived from one-carbon metabolism, is a methyl donor for both DNA and histones methylation^18,19^, while the acetyl-CoA, generated from pyruvate or citrate, is the substrate for histone acetylation^15^.

Mitochondrial metabolic enzymes are also important messengers between mitochondria and nucleus^20^. The TCA cycle enzymes, including oxoglutarate dehydrogenase (OGDH), fumarase (FH1), and pyruvate dehydrogenase (PDH), were reported to translocate into the nucleus where they have roles in DNA repair and epigenetic regulation in some malignant cells^21–23^, indicating that the translocation of metabolic enzymes may be an important pathway for the communication between mitochondria and nucleus^21,24^. However, a role for these types of Spatio-temporal regulations and functions of TCA cycle enzymes in pluripotency acquisition, transition, and totipotency acquisition has not been described. Herein, we show the nuclear translocation of TCA cycle enzymes, especially Pdha1, during the acquisition, transition of pluripotency, and acquisition of totipotency. Nuclear-translocated Pdha1 promotes pluripotency acquisition and transition by increasing the acetyl-CoA pool in the nucleus to promote histone H3 acetylation. Our results reveal the role of TCA cycle enzymes in regulating pluripotency through epigenetic remodeling and provide a model by which mitochondrial-derived factors determine cell fate.

## Results

### Nuclear localization of TCA cycle enzymes during somatic cell reprogramming

**Fig. 1:**
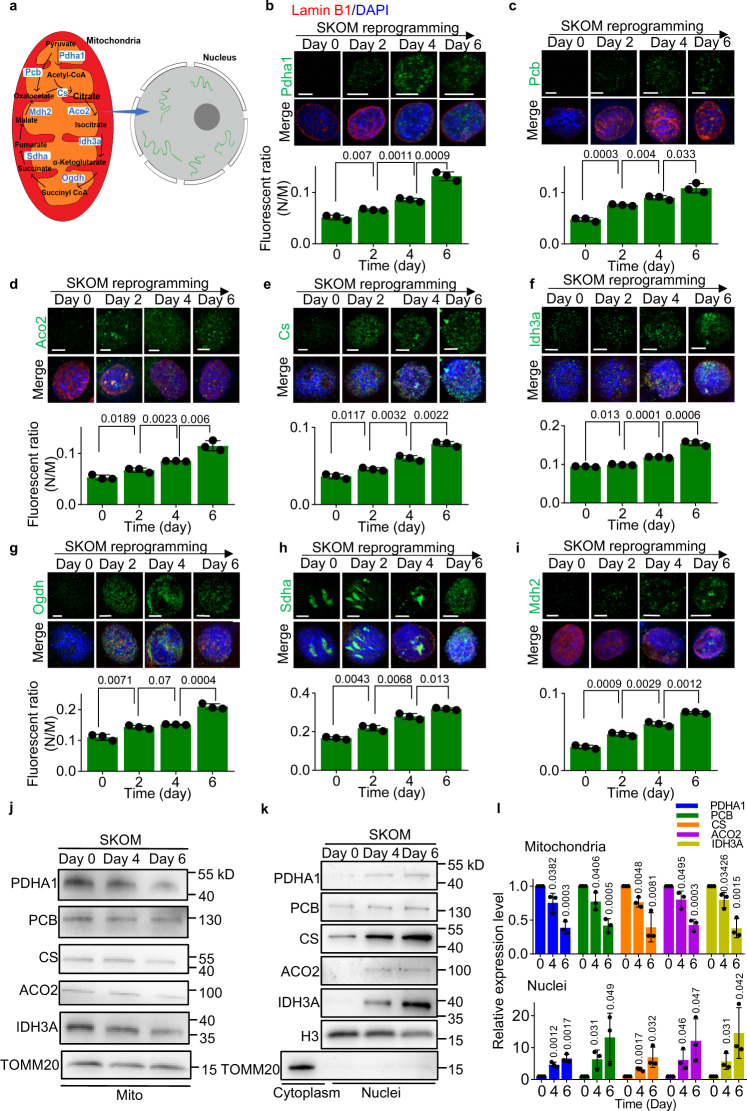
Nuclear localization of TCA cycle enzymes in somatic cell reprogramming. **a** Schematic diagram of TCA cycle enzymes in regulating epigenetics in the nucleus. **b**–**i** Nuclear location of TCA cycle enzymes (green) during the early stages of somatic cell reprogramming with SKOM. Isolated nuclei were stained with antibodies targeting Pdha1 (**b**), Pcb (**c**), Aco2 (**d**), Cs (**e**), Idh3a (**f**), Ogdh (**g**), Sdha (**h**), or Mdh2 (**i**) together with Lamin B1 (red) and DAPI (blue). Scale bars, 5 µm. The nuclei/mitochondria (N/M) fluorescent ratio of each TCA cycle enzyme during somatic cell reprogramming with SKOM present in the source files was shown at the lower panel. Data are presented as the mean ± S.D from three independent experiments containing at least 30 cells each. A two-tailed unpaired Student’s *t*-test was used. **j**, **k**, The western blot analysis of PDHA1, PCB, CS, ACO2, and IDH3A in the mitochondria (**j**) or nucleus (**k**) during somatic cell reprogramming induced by SKOM. The TOMM20 was used as an indicator of mitochondrial content, while H3 were used as loading controls. **l** The quantification of PDHA1, PCB, CS, ACO2, and IDH3A in the mitochondria (upper panel) or nucleus (lower panel) are presented on the right. Data are presented as the mean ± S.D and *n* = 3 independent experiments. A two-tailed unpaired Student’s *t* test was used.

Mitochondrial remodeling and a metabolic switch from OXPHOS to glycolysis are essential to somatic cell reprogramming^8,9^. However, spatio-temporal regulation of and by metabolic enzymes in pluripotency acquisition, primed-to-naive transition, and totipotency acquisition are still barely known (Fig. 1a). Here, we systematically analyzed the cellular location of TCA cycle enzymes at different time points of somatic cell reprogramming induced by SKO (Sox2, Klf4, and Oct4) or SKOM (Sox2, Klf4, Oct4, and c-Myc). Performing immunofluorescence on isolated nuclei, we found that most TCA cycle enzymes, including pyruvate dehydrogenase (Pdha1), pyruvate carboxylase (Pcb), aconitase 2 (Aco2), citrate synthase (Cs), isocitrate dehydrogenase 3 (Idh3a), oxoglutarate dehydrogenase (Ogdh), succinate dehydrogenase (Sdha), and malate dehydrogenase 2 (Mdh2), appeared in the nucleus from the very early stage of somatic reprogramming (Fig. 1b–i and Supplementary Fig. 1b–i), whereas they were highly restricted to the mitochondria in somatic cells (Supplementary Fig. 1a). The fluorescent ratio of these TCA cycle enzymes between nuclei and mitochondria were quantified and these enzymes in the nucleus were increased during reprogramming, comparing to that in the mitochondria (Fig. 1b–i). Among the eight TCA cycle enzymes, Pdha1, Idh3a, Ogdh, and Sdha showed more remarkable nuclear relocation (Fig. 1b, f–h and Supplementary Fig. 1b, f–h) than the other four enzymes, Pcb, Aco2, Cs and Mdh2 (Fig. 1c–e, i and Supplementary Fig. 1c–e, i). We then performed western blot assay, which also detected these enzymes in nucleus fraction during somatic cell reprogramming (Fig. 1j, k). The PDHA1, PCB, CS, ACO2, and IDH3A in the nucleus were gradually increased, while these enzymes in the mitochondria were decreased (Fig. 1j–l). In conclusion, nuclear-localized TCA cycle enzymes could be identified from the early stage of somatic cell reprogramming.

### Nuclear translocation of TCA cycle enzymes in different states of pluripotency

**Fig. 2:**
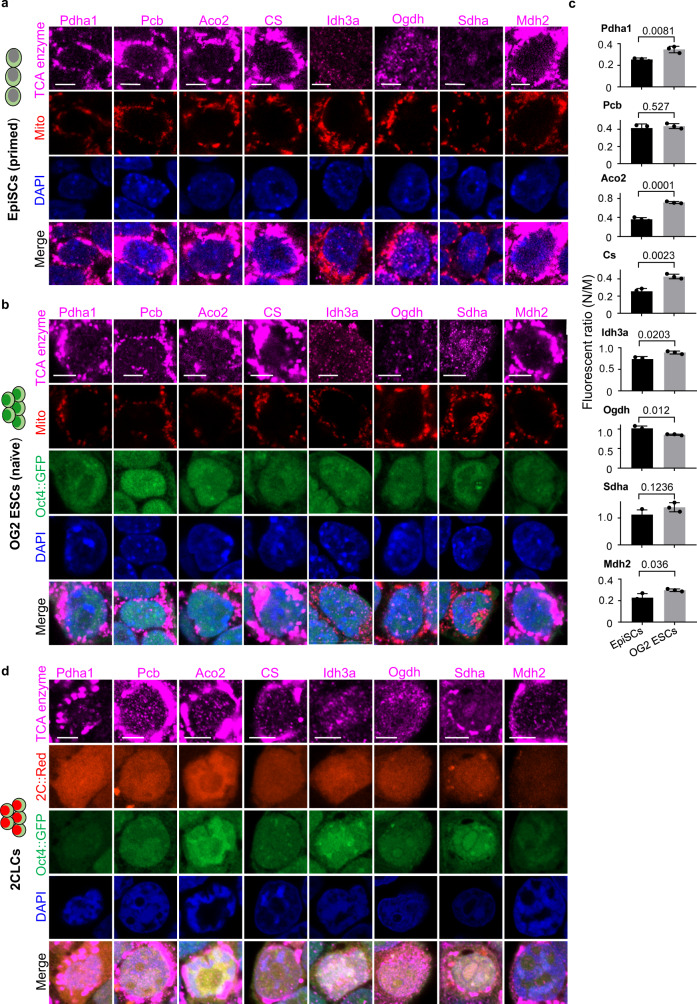
Translocation of TCA cycle enzymes in primed-to-naive transition and totipotency stem cells. **a**, **b** Immunostaining of Pdha1, Pcb, Aco2, Cs, Idh3a, Ogdh, Sdha, and Mdh2 (purple) in mouse epiblast stem cells (EpiSCs) (**a**) or OG2 embryonic stem cells (naive stem cells) (**b**). The mito-DsRed (Mito) was used as a marker of mitochondria. Oct4::GFP is an indicator of embryonic stem cells and DAPI (blue) for indicating the nucleus. Scale bars, 5 µm. **c** The nuclei/mitochondria fluorescent (N/M) ratio of TCA cycle enzymes in EpiSCs and OG2 ESCs (Naive). Data are presented as the mean ± S.D of at least 30 cells from three independent experiments. A two-tailed unpaired Student’s *t* test was used. **d**, Immunostaining of Pdha1, Pcb, Aco2, Cs, Idh3a, Ogdh, Sdha, and Mdh2 (purple) in 2-cell stage-like cells (2CLCs). Oct4::GFP was used to track embryonic stem cells, 2C::tdTomato (red) was used to track 2CLCs, and DAPI (blue) for indicating the nucleus. Scale bars, 5 µm. Three independent experiments were repeated with similar results and one representative picture was presented (**a**, **b**, **d**).

Further, we investigated the localization of these eight TCA cycle enzymes in different states of pluripotent stem cells. The perinuclear distribution of mitochondria was found, while all eight enzymes had a remarkable nuclear localization in both epiblast stem cells (EpiSCs) and naive stem cells (OG2 ESCs) (Fig. 2a, b). What’s more, the nuclei/mitochondria ratio of Pdha1, Aco2, Cs, Idh3a, and Mdh2 were higher in naive ESCs than that in EpiSCs (Fig. 2c). To further study the relationship of nuclear-localized TCA cycle enzymes with totipotency, we detected the localization of these eight TCA cycle enzymes in totipotent stem cells, 2C-like cells (2CLCs), and identified a similar nuclear localization (Fig. 2d). In addition, the molecular weight of TCA cycle enzyme Pdha1 in the nuclear fraction is similar with Pdha1 in mitochondrial fraction and mls-removed Pdha1(Δmls), which was smaller than that of the mitochondrial localization defect mutant (mls^R10,22E^) (Supplementary Fig. 2a–c). This result indicated that the nuclear-localized Pdha1 should also undergo the mls removing process, which occurs in mitochondrial translocation. Meanwhile, overexpression of wild-type Pdha1 showed no effect on the nuclear translocation of Pdha1 (Supplementary Fig. 2d–g). Mitochondrial tethering onto the (Nuclear envelope) NE via Mfn2 could facilitate the release of PDC to the nucleus as reported^25^. The overexpression of Mfn2-NE, which facilitated the tethering of mitochondria to the nucleus, also promoted the nuclear translocation of Pdha1 as well as reprogramming efficiency in our system (Supplementary Fig. 2h–l). All these data indicate a translocation of TCA cycle enzymes to nuclei rather than directly imported to nuclei. Taken together, our results indicate that multiple TCA cycle enzymes translocate to the nucleus during primed-to-naive transition and totipotency acquisition, suggesting roles in pluripotency and totipotency regulation.

### Nuclear-translocated Pdha1 modulates somatic cell reprogramming, primed-to-naive transition, and totipotency acquisition

**Fig. 3:**
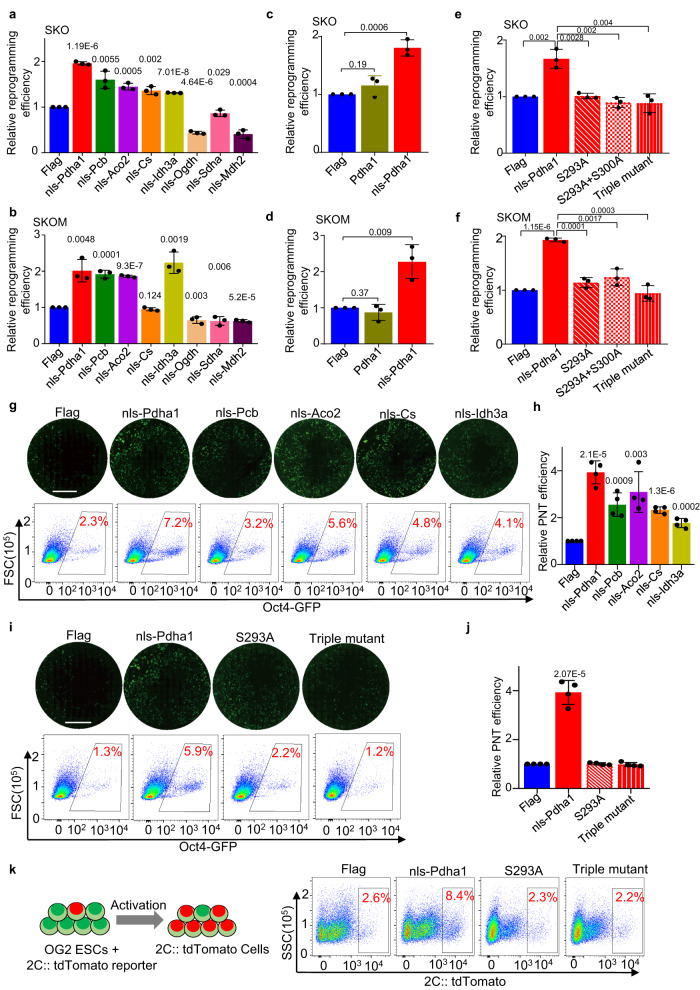
Nuclear TCA cycle enzymes modulate somatic cell reprogramming, primed-to-naive transition and totipotency acquisition. **a**, **b** Relative somatic cell reprogramming efficiency after transducing with nuclear-targeted TCA cycle enzymes together with SKO (**a**) or SKOM (**b**). The reprogramming efficiency was determined by counting the GFP-positive colonies. **c**, **d** The relative somatic cell reprogramming efficiency after overexpression of Pdha1 or nls-Pdha1 together with SKO (**c**) or SKOM (**d**). **e**, **f** The relative somatic cell reprogramming efficiency after overexpression of nls-Pdha1 or nuclear-targeted catalytic mutants of Pdha1-S293A, S293A + S300A or S232A + S293A + S300A (Triple mutant)-together with SKO (**e**) or SKOM (**f**). **g** Representative images (upper panel) and flow cytometry analysis (lower panel) (gated on Oct4-GFP cells) of GFP-positive naive stem cell colonies generated from EpiSCs with nls-Pdha1, nls-Pcb, nls-Aco2, nls-Cs or nls-Idh3a overexpression. The Flag was used as control. Scale bars, 250 µm. Three independent experiments were repeated with similar results. **h** The relative PNT efficiency after overexpression of nls-Pdha1, nls-Pcb, nls-Aco2, nls-Cs, and nls-Idh3a. **i** Representative images (upper panel) and flow cytometry analysis (lower panel) (gated on Oct4-GFP cells) of GFP-positive naive stem cell colonies generated from EpiSCs with nls-Pdha1, S293A, and Triple mutant. Scale bars, 250 µm. Three independent experiments were repeated with similar results. **j** The relative PNT efficiency after overexpression nls-Pdha1, nls-Pdha1, S293A and Triple mutant. **k** Left: Schematic of OG2 ESCs with 2C:: tdTomato reporter induced into totipotency stem cells. Right: Flow cytometry (gated on 2C:: tdTomato cells) showing the relative proportion of 2C:: tdTomato-positive totipotency stem cells generated from OG2 ESCs with overexpression of nls-Pdha1 or nuclear-targeted catalytic mutants of Pdha1 (right). Data are presented as the mean ± S.D (**a**–**f**, **h**, **j**), and *n* = 3 independent experiments (**a**–**f**) or *n* = 4 independent experiments (**h**, **j**). A two-tailed unpaired Student’s *t* test was used (**a**–**f**, **h**, **j**).

To test whether translocated TCA cycle enzymes play roles in somatic cell reprogramming, we replaced the mitochondrial localization signals (MLS) of each of these enzymes with a nuclear localization sequence (NLS) and 3$\times$Flag tag (Supplementary Fig. 3a). These modified enzymes—nls-Flag-Pdha1, nls-Flag-Aco2, nls-Flag-Pcb, nls-Flag-Cs, nls-Flag-Idh3a, nls-Flag-Ogdh, nls-Flag-Sdha, and nls-Flag-Mdh2—were observed in nucleus (Supplementary Fig. 3a, b). Then we tested their effects on SKO and SKOM-mediated somatic cell reprogramming and showed a remarkable increase in reprogramming efficiency upon overexpression of the nuclear-translocated TCA cycle enzymes, Pdha1, Pcb, Aco2, Cs, and Idh3a, but not by Ogdh, Sdha, and Mdh2 (Fig. 3a, b). Among the nuclear-translocated TCA cycle enzymes, nls-Pdha1 showed the greatest efficiency in promoting both SKO and SKOM reprogramming (Fig. 3a, b).

Notably, overexpression of wild-type Pdha1 had little effect on SKO-/SKOM- mediated reprogramming efficiency (Fig. 3c, d), highlighting the potential of a role for nuclear translocation of Pdha1 in promoting reprogramming. Thus, we focused on Pdha1, an important subunit of the PDH complex which converts pyruvate to acetyl-CoA^26^. As the S- > A point mutations in the catalytic subunit of nls-Pdha1 at S232, S293 and S300 could inactive Pdha1^27^, we introduced these point mutations in nls-Pdha1, which showed neither effect on the nucleus localization (Supplementary Fig. 3c), or gene expression (Supplementary Fig. 3d–f). The mutations could also abrogate the effect on somatic cell reprogramming of nls-Pdha1 (Fig. 3e, f), indicating the promotion of reprograming by nuclear-translocated Pdha1 is dependent on its catalytic activity. To further investigate the time window of nls-Pdha1’s effect on reprogramming, we expressed nls-Pdha1 under the control of a doxycycline (Dox)-inducible promoter. The overexpression of nls-Pdha1 by Dox addition promoted reprogramming at all tested intervals, but had greatest effect on iPSC generation was observed by Dox treatment throughout days 1-15, indicating nls-Pdha1 functions at the whole process of reprogramming (Supplementary Fig. 4a). The iPSC colonies were characterized by the expression of pluripotency genes and three germ-line differentiation (Supplementary Fig. 4b–f). These results suggested that the promotion of reprogramming by nuclear-localized Pdha1was reliant on the catalytic activity.

We then asked whether the translocated TCA cycle enzymes played roles in the primed-to-naive transition (PNT) of PSCs (Fig. 3g). A remarkable increase in PNT efficiency was identified upon overexpression of nls-Pdha1, nls-Pcb, nls-Aco2, nls-Cs and nls-Idh3a (Fig. 3g, h). It is the same for the activation of the naive markers, such as *Rex1*, *Esrrb*, *Nanog*, *Stella*, *Dppa3*, *Dppa4* and *Dppa5*, as well as the suppression of primed markers, including *Fgf5*, *Fgf8,* and *T* (Supplementary Fig. 4g). Notably, nls-Pdha1 showed the greatest efficiency in promoting PNT process (Fig. 3g, h). However, the point mutations of Pdha1 in the catalytic subunit, S293A and Triple mutant, lost the ability in PNT as predicted (Fig. 3i, j). The overexpression of nls-Pdha1 promoted the naive marker genes’ expression as well as the proportion of naive cells, and inhibit the primed marker genes’ expression in PNT process (Supplementary Fig. 4h). To further investigate the roles of nuclear-translocated Pdha1 in the totipotency acquisition, we overexpressed nls-Pdha1 in OG2 ESCs with a 2C reporter, and found it could increase the proportion of 2CLCs, while the mutants in catalytic subunit lost the ability (Fig. 3k). These results indicated that nuclear translocation of Pdha1 plays important roles in both somatic cell reprogramming, primed-to-naive transition and totipotency acquisition which is dependent on its catalytic subunit.

### Nuclear-translocated Pdha1 increases the metabolic pool in the nucleus

**Fig. 4:**
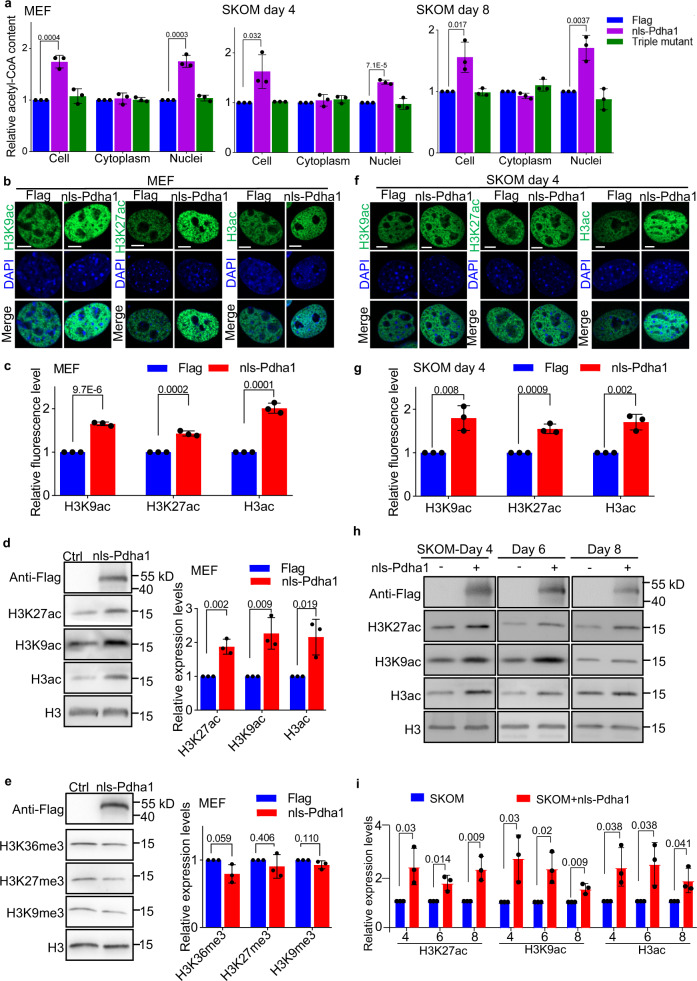
Nuclear Pdha1 promotes histone acetylation by increasing the local acetyl-CoA content. **a** Relative content of acetyl-CoA in whole-cell, cytoplasm, or nucleus of MEFs during somatic cell reprogramming with SKOM. Cells after overexpression of Flag, nls-Pdha1, or nls-Pdha1 with triple mutation of its catalytic domain (Triple mutant) were tested. **b**, **c** Representative images (**b**) and quantification (**c**) of H3K9ac, H3K27ac, and H3ac (green) in MEFs with or without nls-Pdha1 overexpression. Scale bars, 5 µm. **d** Western blot analysis of H3K9ac, H3K27ac, and H3ac in MEFs with or without nls-Pdha1 overexpression, as well the band quantification. Anti-Flag was targeting nls-Pdha1 and Anti-H3 was used as a loading control. **e** H3K9me3, H3K27me3, and H3k36me3 modifications in MEFs with or without nls-Pdha1 overexpression were detected by western blot. Anti-Flag was targeting nls-Pdha1 and Anti-H3 was used as a loading control. The relative expression levels were quantified in the right panel. **f**, **g** Representative images (**f**) and quantification (**g**) of H3K9ac, H3K27ac, and H3ac (green) at day 4 of cell reprogramming with SKOM plus nls-Pdha1 or Flag (control). Scale bars, 5 µm. **h** The H3K9ac, H3K27ac, and H3ac modifications during cell reprogramming with or without nls-Pdha1 at indicated time point were detected by western blot. Anti-Flag was targeting nls-Pdha1 and Anti-H3 was used as a loading control. **i** The relative expression levels of H3K9ac, H3K27ac, and H3ac modifications during cell reprogramming with or without nls-Pdha1 at each time point were quantified. Data are presented as the mean ± S.D and *n* = 3 independent experiments (**a**, **c**–**e**, **g**, **h**, **i**). At least 30 cells were counted in each experiment (**c**, **g**). A two-tailed unpaired Student’s *t* test was used (**a**, **c**–**e**, **g**, **i**).

The catalytic activity of nuclear-translocated Pdha1 may be critical for cell fate determination, as mutations in the catalytic domain of nls-Pdha1 eliminated its ability to promote reprogramming (Fig. 3e, f). To further test how nls-Pdha1’s catalytic function is involved, we measured the acetyl-CoA content of the cytoplasm and nucleus of MEFs during reprogramming induced by SKOM. Overexpression of nls-Pdha1 increased acetyl-CoA content in the nucleus without affecting cytoplasmic acetyl-CoA, while expression of catalytic domain mutant (Triple mutant) of nls-Pdha1 had little effect on acetyl-CoA in the nucleus (Fig. 4a). The other two acetyl-CoA-generating enzymes ATP citrate lyase (Acly) and acyl-CoA synthetase short-chain family, member 2 (Acss2)^26,28^ also showed nuclear localization both in MEFs and somatic cell reprogramming process (Supplementary Fig. 5a–c). Overexpression of the nuclear-targeted Acly or Accs2 by adding a nls on the N-terminal could also induce nuclear acetyl-CoA production and promote reprogramming, as nls-Pdha1 (Supplementary Fig. 5d, e). Moreover, the content of pyruvate, citrate and α-KG in the nucleus was also increased with nls-Pdha1 overexpression, but not by the Triple mutant (Supplementary Fig. 6a–c). Then we asked whether nls-Pdha1 alters cellular bioenergetics. No significant differences in OCR (oxygen consumption rate) or ECAR (extracellular acidification rate) were observed between MEFs undergoing reprogramming with and without nls-Pdha1 overexpression (Supplementary Fig. 6d–g). Thus, these data indicate that the nucleus-localized Pdha1 increases the local metabolic pool in the nucleus and does not affect overall cellular bioenergetics.

### Nucleus-localized Pdha1 promotes histone H3 acetylation

As acetyl-CoA in nucleus is a critical substrate for histone acetylation^15^, we asked whether histone acetylation was affected by nls-Pdha1. We performed immunofluorescence staining assay in MEFs and found that histone H3 acetylation, including H3K9ac, H3K27ac, and the total acetylation of H3 (H3ac), increased upon nls-Pdha1 overexpression, but not by Triple mutant (Fig. 4b, c and Supplementary Fig. 7a). Western blot analysis also showed increased histone acetylation in nls-Pdha1 overexpressing cells (Fig. 4d and Supplementary Fig. 7b) but no significant differences in histone methylation (Fig. 4e). To further confirm this, we examined histone acetylation in cells undergoing reprogramming with or without nls-Pdha1. We also observed a significant increase in H3 acetylation, including H3K9ac, H3K27ac, and H3ac, in reprogramming at days 4 and 6 upon nls-Pdha1 overexpression (Fig. 4f–i and Supplementary Fig. 7c, d).

**Fig. 5:**
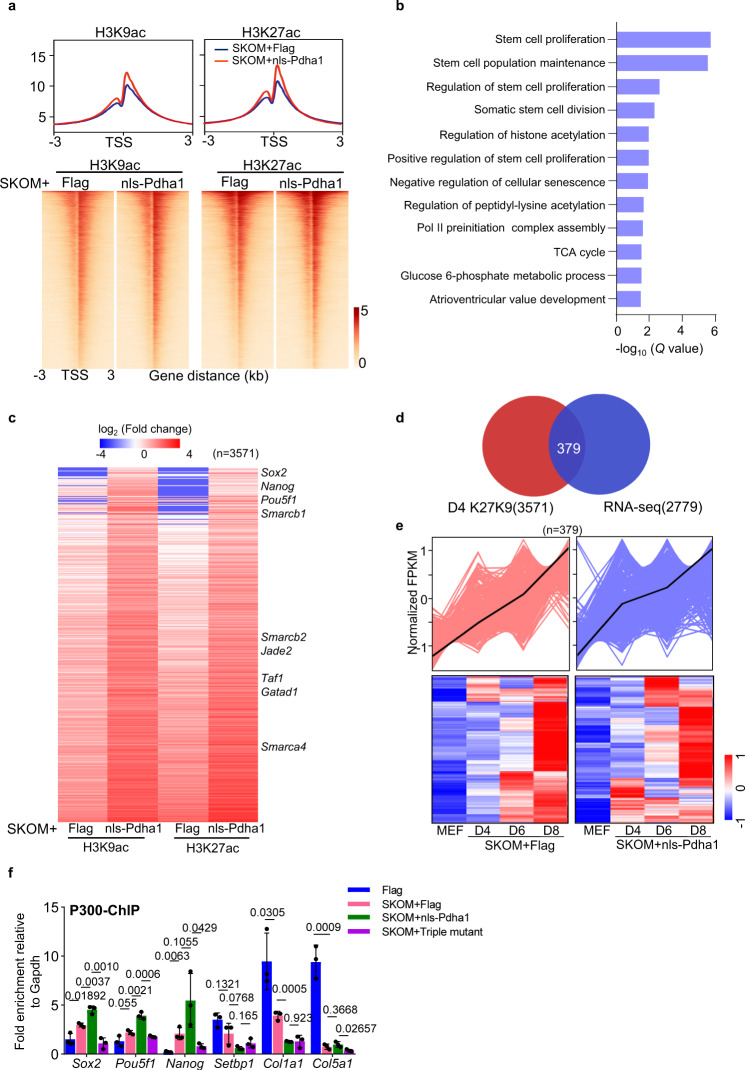
Nuclear-localized Pdha1 promotes histone acetylation of pluripotent genes by facilitating the enrichment by P300. **a** Pileup (upper panel) and heatmap (lower panel) of H3K9ac, H3K27ac ChIP-seq near the TSS in MEFs transduced with SKOM plus nls-Pdha1 or Flag on day 4. 2 independent experiments were repeated with similar results, and one replicate (*n* = 1, each group) was used for ChIP-seq (upstream 3 kb and downstream 3 kb of the TSS). **b** GO analysis for the genes with >1.5-fold enrichment of H3K9ac and H3K27ac in the nls-Pdha1 overexpression group at day 4 during somatic cell reprogramming. **c** Global view of H3K9ac and H3K27ac levels at genes with >1.5-fold upregulated normalized tag density near the TSS at day 4. A total of 3571 genes were contained. Two independent experiments were repeated with similar results, and one replicate (*n* = 1, each group) was used for this analysis. **d** Venn diagram depicting overlap of the genes where both H3K9ac and H3K27ac increased around TSS sites with genes upregulated in somatic cell reprogramming. A total of 3571 genes were contained in ChIP-seq data, while a total of 2779 genes were contained in RNA-seq data. **e** Dynamic of the overlayed genes (**d**) in SKOM induced reprogramming with or without nls-Pdha1 or Flag. 2 independent experiments were repeated with similar results, and one replicate (*n* = 1, each group) was used for this analysis (**b**–**e**). **f** ChIP-qPCR analysis of P300 enrichment on promoter regions of pluripotency genes (*Sox2*, *Pouf51*, *Nanog*) and somatic genes (*Setbp1*, *Col1a1*, *Col5a1*). Data are presented as the mean ± S.D and *n* = 3 independent experiments. A two-tailed unpaired Student’s *t* test was used.

To further investigate the epigenetic regulation by nuclear-translocated Pdha1, we performed chromatin immunoprecipitation sequencing (ChIP-seq) during reprogramming in cells with or without nls-Pdha1 overexpression. The distribution of H3K9ac or H3K27ac on cis-elements were not affected by nls-Pdha1 overexpression (Supplementary Fig. 7e, f). However, the H3K9ac signal and H3K27ac signal around transcription start sites (TSS) was increased remarkably upon nls-Pdha1 overexpression in reprogramming at days 4 and 6 (Fig. 5a and Supplementary Fig. 7g, h). The similar effect were identified on enhancers upon nls-Pdha1 overexpression (Supplementary Fig. 7i, j). Gene ontology (GO) term analysis indicated that the set of upregulated genes is enriched in genes involved in stem cell proliferation and stem cell population maintenance (Fig. 5b). Then, we collected all loci enriched for H3K9ac and H3K27ac at transcription start sites (TSS) and pulled out genes where both H3K9ac and H3K27ac increased significantly (fold change>1.5) after nls-Pdha1 overexpression at day 4 of reprogramming. The gene list with higher enrichment of H3 acetylation upon nls-Pdha1 overexpression includes many chromatin remodelers and pluripotency genes such as *Smarcb1*, *Pou5f1* (*Oct4*), *Nanog* (Fig. 5c). To explore the details in epigenetic regulation by nls-Pdha1 in somatic cell reprogramming, we overlayed the H3K9ac and H3K27ac enriched genes after nls-Pdha1 overexpression during somatic cell reprogramming with the upregulated genes in somatic cell reprogramming (Fig. 5d), and identified a subset of 379 overlayed genes, which showed a shift in the dynamic of genes’ expression with a gene activation earlier after nls-Pdha1 overexpression than the control group during somatic cell reprogramming (Fig. 5e). However, no signal was detected in ChIP assay of Pdha1 (Supplementary Fig. 8a-d). Meanwhile, the ChIP analysis of P300, a key regulator in histone acetylation, showed greater enrichment around promoters of pluripotency-related genes (*Sox2*, *Pou5f1*, *Nanog*) than somatic genes (S*etbp1*, *Col1a1*, *Col5a1*) during reprogramming, suggesting pluripotency genes may be more sensitive to acetyl-CoA levels (Fig. 5f). The increased enrichment of P300 in the promoter region of pluripotency genes was also observed after nls-Pdha1 overexpression, but not Triple mutant overexpression (Fig. 5f). We also carried on ChIP-qPCR to assess the binding of the Yamanaka factors (*Sox2*, *Klf4,* and *Oct4*) to pluripotency genes in reprogramming and identified that *Sox2*, *Klf4,* and *Oct4* bound to pluripotency genes more readily after co-expressing with nls-Pdha1, but not Triple mutant (Supplementary Fig. 8e). Taken together, these data suggested that nucleus-localized Pdha1 improves the histone acetylation on a series of chromatin remodelers and pluripotency-related genes.

### Nuclear-translocated Pdha1 loosens chromatin of pluripotency genes

**Fig. 6:**
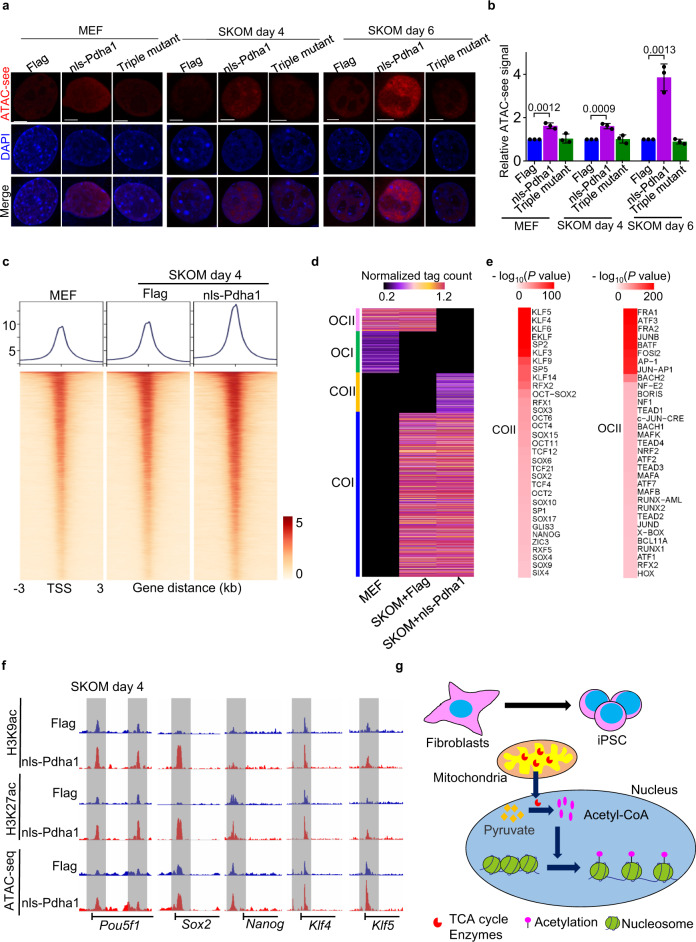
Nuclear Pdha1 loosens chromatin of pluripotency genes and promotes somatic cell reprogramming. **a** Representative images of ATAC-see (red) in MEFs before and after transduction with SKOM plus nls-Pdha1, Triple mutant or Flag at day 4 and day 6. Scale bars, 5 µm. **b** Quantification of the relative levels of ATAC-see signal represented in **a**. Data are presented as the mean ± S.D and *n* = 3 independent experiments. At least 30 nuclei were counted in each experiment. A two-tailed unpaired Student’s *t* test was used. **c** Heatmap and pileup of ATAC-seq signal near the TSS (upstream 3 kb and downstream 3 kb of the TSS) in MEFs and MEFs transduced with SKOM plus nls-Pdha1 or Flag on day 4. Three replicates (*n* = 3, each group) were used for the ATAC assay. **d** Heatmap of ATAC-seq OC/CO loci at day 4 during reprogramming: closed in Flag but open in Flag+SKOM and nls-Pdha1+SKOM (COI), closed in Flag and Flag+SKOM but open in nls-Pdha1+SKOM (COII), open in Flag but closed in SKOM + Flag and SKOM + nls-Pdha1 (OCI), open in Flag and SKOM + Flag but closed in SKOM + nls-Pdha1 (OCII). Units are in normalized sequence tag counts. Three replicates (*n* = 3, each group) were used for ATAC assay. **e** TF motifs significantly enriched at least >1.5-fold in COII (left) and OCII (right) peaks defined in **d**. The motif enrichment was conducted using the findMotifsGenome.pl module in HOMER. Three independent experiments were repeated with similar results, and three replicates (*n* = 3, each group) were used for this analysis. **f** Selected genome views for H3K9ac and H3K27ac ChIP-seq data and ATAC-seq in reprogramming on day 4 with or without expression of nls-Pdha1. Regions of open chromatin are marked with a gray box. Genome views: *Nanog* (chr6:122,704,483–122,716,633), *Pou5f1* (chr17:35,504,032–35,512,777), *Klf4* (chr4:55,525,137–55,534,475), *Sox2* (chr3:34,647,995–34,654,461) and *Klf5* (chr14:99,296,691–99,315,412). All genome views are on the same vertical scale. **g** Schematic diagram represents the mechanism of nuclear translocation of Pdha1 in regulating cell fate through epigenetic regulation.

As H3 acetylation is an important epigenetic modification of chromatin, we then asked how nls-Pdha1 remodels chromatin. We monitored chromatin opening status using transposase-accessible chromatin with visualization (ATAC-see) methods, in which ATAC-see probe signal indicates chromatin opening status^29^. As expected, a higher level of ATAC-see signal was observed upon nls-Pdha1, but not by the Triple mutant overexpression in both naive MEFs and cells undergoing SKOM reprogramming (Fig. 6a, b). To further identify the loosened gene loci in reprogramming upon nls-Pdha1 overexpression, we performed ATAC-seq (Fig. 6c and Supplementary Fig. 9a). We pulled out the peaks of open chromatin by comparing the loci between MEFs and cells undergoing reprogramming. These peaks were classified as closed in MEFs but open in reprogramming cells (CO) or open in MEFs but closed in reprogramming cells (OC) as in our previous report^30^. Similarly, the accessible and closed gene loci specific to nls-Pdha1 overexpression in reprogramming were defined as COII and OCII (Fig. 6d and Supplementary Fig. 9b, c). A series of pluripotency-related genes such as *Klf5*, *Sox2*, *Nanog,* and *Oct4* were identified at COII peaks with ATAC-seq upon nls-Pdha1 overexpression, while many somatic-related genes, such as *Fra1*, *Arf3*, *Ap1,* and *Tead3*, were identified to be enriched at OCII peaks (Fig. 6e). GO analysis of the COII genes represented that the genes with accessible chromatin in SKOM plus nls-Pdha1 were associated with stem cell population maintenance, regeneration, and stem cell proliferation (Supplementary Fig. 9d). The genome views for key pluripotency genes (*Nanog, Pou5f1, Klf4, Sox2, and Klf5*) showed increased peaks with nls-Pdha1 overexpression at day 4 during reprogramming (Fig. 6f). A strong correlation was also identified between COII genes’ peaks and H3K9ac/H3K27ac modifications. Importantly, loci around TSS of pluripotency genes such as *Nanog, Pou5f1, Klf4, Sox2, and Klf5* were opened upon nls-Pdha1 overexpression, accompanied by higher level of H3K9ac and H3K27ac (Figs. 6f and 5c). These data indicate that nucleus-localized Pdha1 leads to remodeling of chromatin structure around pluripotency genes.

## Discussion

We have shown the translocation of TCA cycle enzymes to the nucleus in somatic cell reprogramming, primed-to-naive transition and totipotency acquisition (Fig. 6g). Translocation of a subset of TCA cycle enzymes as Pdha1, Aco2, Pcb, Cs and Idh3a to nucleus promoted the processes of pluripotency acquisition and transition. Nucleus-targeted Pdha1 increased the nuclear acetyl-CoA pool leading to increased histone H3 acetylation. This effect was abrogated by disabling mutations in the catalytic domain of nuclear Pdha1^21^. Further, increased H3K9ac and H3K27ac remodeled the chromatin structure of pluripotency genes and promoted their expression. Our study reveals the importance of mitochondria-to-nucleus translocation of TCA cycle enzymes in regulating pluripotency through epigenetic remodeling, which represents a retrograde signaling mechanism in cell fate determination.

While it has been known that intermediate products of TCA cycle are important substrates for epigenetic modification^31,32^, we showed that the TCA cycle enzymes of mitochondria such as Pdha1 could also relocate to the nucleus and regulate chromatin modifications (Fig. 6g). Embryonic stem cells largely rely on glycolysis for energy production and their mitochondria lack crista and are not fully functional^33,34^. However, more and more evidence show that mitochondria play important roles in regulating the epigenetics and pluripotency of stem cells^7,35^. Our model illustrates roles of mitochondria in the epigenetic regulation of stem cell fate determination. The relocation of TCA cycle enzymes could also be identified in 2-cell stage-zygotes of mouse embryos^24^, which further supports the idea that mitochondria take part in the cell fate determination via TCA cycle enzymes relocation in the early stage of embryonic development.

Nuclear translocation of mitochondrial metabolic enzymes is a type of mitochondria-to-nucleus retrograde signaling in epigenetic modification. Many metabolites generated by mitochondria, such as NAD^+^, SAM, α-KG, acetyl-CoA, are well-known substrates for epigenetic modification^31^. More and more epigenetic modifications have been identified, such as histone palmitoylation, crotonylation, and butyrylation, which also rely on metabolites generated by mitochondria^17,36^. Similar to the nuclear translocation of Pdha1 and other TCA cycle enzymes we described here, OGDH has been reported to translocate to the nucleus and regulate the histone succinylation in U251 cells^23^. Besides Pdha1 we investigated, other translocated TCA cycle enzymes, Pcb, Aco2, and Cs, may also play similar roles in regulating epigenetics in the nucleus. A recent report identified a TCA cycle in the nucleus similar to that in mitochondria^37^, which is in line with our report and it would be a pathway in epigenetic regulation.

Pluripotent stem cells possess an open chromatin structure, which is critical for pluripotency^38,39^. Hyperactive histone acetylation is critical for maintaining the open chromatin status of PSCs^40^. Previous reports suggested that aerobic glycolysis is one of the important sources of acetyl-CoA for histone acetylation^15^. Here, we propose another pathway, in which nuclear translocation of Pdha1 fuels the local acetyl-CoA pool for histone acetylation in stem cells. Nuclear translocation of Pdha1 was observed in the early stages of somatic cell reprogramming, and in both primed stem cells and naive stem cells, indicating the importance of nuclear metabolic enzymes in maintaining the hyperactive histone acetylation in PSCs. The nuclear translocation of other TCA cycle enzymes in pluripotent stem cells also supports the idea that this is a unique pathway in pluripotency acquisition and primed-to-naive transition. Cancer stem cells (CSCs) also exhibit open chromatin structure, hyperactive histone acetylation, and metabolic switch from oxidative phosphorylation to aerobic glycolysis; our findings linking nuclear translocation of TCA cycle enzymes to epigenetic regulation thus may also inform research into CSCs^41,42^.

There are few reports about the mechanism of translocation of TCA cycle enzymes. Nagaraj et al.^24^ proposed that a fraction of TCA cycle enzymes could translocate from mitochondria to the nucleus through O-linker glycosylation with the help of Hsp70 or Hsp90 in the 2-cell stage. A recent study also suggested that PDHE1α could translocate from cytosolic to mitochondria in peritumoral tissues after being phosphorylated by ERK2^43^. The mitochondrial PDC complex could also be translocated to the nucleus by the tethering of mitochondria and the nucleus with the help of MFN2 as reported^25^. However, the mystery of how the mitochondrial matrix localized PDC complex across both mitochondrial inner membrane and nuclear membrane, and translocated to the nucleus is still not fully covered. Whether the TCA cycle enzymes translocated from mitochondria to the nucleus in the embryonic stem cell by the same pathway is worthy to study. The ESCs possess more naive mitochondria, which lack cristae structure, and are perinuclear localized (Fig. 2a, b), which may be an important advantage for the translocation of TCA cycle enzymes. Meanwhile, the nuclear envelope is also rapidly reconstructed in stem cells as the fast division, which may also be helpful for the translocation of both TCA cycle enzymes and metabolites.

## Methods

### Cell culture

OG2 MEFs were derived from E13.5 embryos of OG2 mice, carrying the Oct4-GFP transgenic allele as reported^30^. The same for OG2 mESCs or EpiSCs, which were derived from cells of E3.5 or E5.5 embryos, respectively. ICR MEFs were derived from E13.5 embryos of ICR mice carrying the Oct4-GFP transgenic allele. OG2 MEFs, ICR MEFs, Plat-E (ATCC; RRID: CVCL-0063), HEK293T (ATCC, catalog no. CRL-3216), and Hela (ATCC, catalog no. CCL-2) cells were maintained in DMEM/high glucose (Gibco, 11995500BT) supplemented with 10% FBS (Gibco, 10099141), 1% GlutaMAX (Gibco, 35050079) and 1% NEAA (Gibco, 1140076). OG2 mESCs were cultured on plate precoated with 0.1% gelatin and maintained with DMEM/high glucose supplemented with 0.5% N2 (Gibco, 17502048), 1% B27 (Gibco, 17504044), 1% GlutaMAX, 1% NEAA, 1% sodium pyruvate (Gibco, 11360070), 0.1 mM β-mercaptoethanol (Gibco, 21985-023), 1000 U/mL leukemia inhibitory factor (LIF), 3 μM CHIR99021 (Selleck, S1263) and 1 μM PD0325901 (Selleck, S1036). The cells were obtained with approval from the ethics committee of the Guangzhou Institutes of Biomedicine and Health, Chinese Academy of Sciences (GIBH).

### DNA construction

The pMXs vectors expressing Sox2, Klf4, Oct4, or c-Myc were purchased from Addgene. The mouse Pdha1(28-390), Pdha1 mutants (28-390), Pcb (22-1179), Aco2 (28-780), Cs (26-464), Idh3a (26-366), Ogdh (40-1038), Sdha (40-664) and Mdh2 (40-388) fusion with a SV40 NLS sequence (PKKKRKV) after removing MLS were cloned into pMXs vector or pRlenti-EF1α−2A-Puro vector. The full-length Acly and Acss2 fused with SV40 NLS sequence were cloned into the pMXs vectors, and the same for the mitochondria localization sequence deletion mutant (Δmls) and the mls point mutant (mls^R10,22E^) of Pdha1. Inducible nls-Pdha1 was cloned into a pW-TetOn vector. The plasmids were listed in Supplementary Table 1.

### Somatic cell reprogramming

Retroviral vectors expressing mouse Sox2, Klf4, Oct4, and c-Myc (SKOM) were transfected into Plat-E cells using polyethyleneimine (PolyScience, 23966-1) to produce retrovirus. A total of 1.5 × 10^4^ OG2 MEFs within two passages were seeded in 12-well plate and infected twice with retroviral supernatants of SKO or SKOM. The cells were further cultured in mES medium containing 82% DMEM/high glucose, 15% FBS (Gibco, 10099141), 1% sodium pyruvate, 1% GlutaMAX, 1% NEAA, 0.1 mM β-mercaptoethanol (Gibco, 21985-023), 1000 U/mL leukemia inhibitory factor (LIF). Oct4-GFP-positive colonies were counted on day 17 after infection. The reagent used were listed in Supplementary Table 2.

### Induction of EpiSCs to naive state (PNT)

The induction of PNT was performed following the previous report^44^. Lentivirus vectors coding nuclear-targeted TCA cycle enzymes were transfected into HEK293T cells using polyethyleneimine to produce lentivirus. A total of 2.5 × 10^4^ EpiSCs were seeded in a 12-well plate and infected twice with lentivirus containing nuclear-targeted TCA cycle enzymes. The cells were cultured in N2B27 + XAV939 + FA medium with the addition of Y27632 after treatment with 1 μg/ml puromycin for 2 days. The medium was replaced with mES + 2i/LIF + doxycycline the next day and further cultured for another 5 days. The medium was changed daily. The ingredients of FA medium and N2B27 medium were the same as reported^44,45^.

### The activation of 2-cell-like program

The OG2 ES cells with 2C::tdTomato reporter were generated as the previous report^46^. A total of 3 × 10^4^ reporter cells were seeded in six-well plate and infected twice with lentivirus containing nls-Pdha1, S293A, or (S232A + S293A + S300A) (Triple mutant). The cells were induced by changing with mES + 2i/LIF + doxycycline medium after treatment with 1 μg/ ml puromycin for 2 days.

### Karyotype and teratoma formation

The karyotype was performed as in our previous report^47^. Briefly, the nls-Pdha1-iPSCs at a confluence of 60-70% were used for karyotype analysis after treatment with, 0.02 mg/ml demecolcine (Enzo Life Sciences, LKT-D1749-M050) for 1 hour. The cells were washed, trypsinized, pelleted, and resuspended in 0.075 M KCl and collected by centrifugation (300 ×* g*) after fixing with cold 3: 1 methanol: acetic acid) for 10 minutes at 37 °C. After dropping on a cold slide and stained with Giemsa stain (Coolaber, SL7010), the karyotype was detected.

For teratoma formation, female BALB/c-Nude mice (6–8-weeks old) were purchased from GemPharmatech Co., LTD (Nanjing, China). About 2 × 10^6^ nls-Pdha1-iPSCs were injected into the dorsal flank after suspending at 1 × 10^7^ cells/ml in DMEM containing 50% matrigel. Tumors were fixed with paraformaldehyde (PFA) after surgically dissecting from the mice ~4-6 weeks after the injection. After embedding in paraffin, teratoma samples were sectioned and stained with hematoxylin and eosin (Abcam, ab245880). All animals were handled following the guidelines of the National Institutes of Health for the Care and Use of Laboratory Animals (NIH Publications No. 8023, revised 1978). the experiments were performed according to the protocol N2022106 approved by the Institutional Animal Care and Use Committee (IACUC) of Guangzhou Institutes of Biomedicine and Health. All mice have free access to food and water and were housed in a pathogen-free environment with a 12:12 dark/light cycle, controlled temperature (23 ± 2 °C) and humidity (60 ± 10%).

### Quantitative PCR (QPCR)

Total RNA of cells was extracted by RNA Purification kit (EZBioscience, B0004DP) according to the manufacturer’s protocol, and 1 μg RNA was used to generate cDNA with a Reverse Transcription kit (Takara, RR047A). The PCR reaction was run on a Quantitative PCR machine (Bio-Rad, CFX96) with an SYBR Green QPCR kit (TaKaRa, RR820A). The primers used are listed in Supplementary Table 6.

### Western blots

Cells were lysed with RIPA (Beyotime, P0013B) containing 1 mM PMSF (Beyotime, ST506) and protease inhibitor cocktail (Roche, 04693116001). Nuclear protein was isolated following the manual of the Nuclear and Cytoplasmic Protein Extraction kit (Beyotime, P0028). The membranes after protein transfer were incubated with antibodies overnight at 4 °C. After washing with TBST, the membranes were incubated with secondary antibodies conjugated to HRP at room temperature for 1.5 h. ECL solution (Millipore, WBKLS0500) was added and signals were detected using a Mini Chemi910 Chemiluminescent/Fluorescent Imaging and Analysis System (SageCreation, Beijing, China). The anti-H3 (Abcam, ab1791, 1:5000), anti-H3K9ac (Cell Signaling Technology, 9649, 1:3000), anti-H3K27ac (Cell Signaling Technology, 8173, 1:3000), anti-H3ac (Abcam, ab47915, 1:3000), anti-H3K9me3 (Abcam, ab8898, 1:3000), anti-H3K27me3 (Abcam, ab272165, 1:3000), anti-H3K36me3 (Abcam, ab194677, 1:3000), anti-Tomm20 (Abcam, ab56783, 1:3000), anti-Pdha1 (Abcam, ab110334, 1:1000), anti-Pcb (Abcam, ab229267, 1:1000), anti-Aco2 (Abcam, ab110321, 1:1000), anti-Idh3a (Abcam, ab228596, 1:1000), anti-Cs (Abcam, ab966000, 1:1000), anti-Flag (Sigma, F1804, 1:2000), HRP-conjugated goat anti-mouse antibody (Kangchen, KC-MM-035, 1:4000), HRP-conjugated goat anti-rabbit antibody (Kangchen, KC-RB-035, 1:4000) antibodies were used. The antibodies used are listed in Supplementary Table 5.

### OCR and ECAR analysis

The oxygen consumption rate (OCR) and extracellular acidification rate (ECAR), are indicators of mitochondrial respiration and glycolysis, respectively (Seahorse Biosciences). A total of 8 × 10^4^ cells were plated on gelatin-coated plates overnight at 37 °C. OCR and ECAR were determined with the Seahorse XF Cell Mito Stress Test Kit (Agilent Technologies, 103015-100) and Glycolysis Stress Test Kit (Agilent Technologies, 103020-100), respectively. For OCR measurement, 2 μM oligomycin, 1 μM FCCP, 1 μM rotenone, and 1 μM antimycin supplied by the kit was used. For ECAR measurement, 10 mM glucose, 2 μM oligomycin, and 100 mM 2-deoxyglucose supplied by the kit were used.

### Immunofluorescence

Cells were fixed 24 hours after seeding on the coverslips with 4% paraformaldehyde (PFA) for 10 minutes, and permeabilized with 0.5% Triton X-100 (Sigma, T8787) for 10 minutes. The cells were incubated with primary antibodies at indicated dilution overnight at 4 °C after blocking with 10% FBS for 1 hour at room temperature. After washing with PBS for five times, the slides were incubated with either anti-rabbit or anti-mouse antibodies conjugated with Alexa Fluor 488, 568, and 647 for 1 hour at room temperature. The slides were mounted after staining with 1 μg/ml DAPI (Sigma, D9542) for 10 minutes using a mounting medium (DAKO, S3023). Imaging was carried out on a Zeiss LSM 880 or Zeiss LSM 800 confocal microscope. The anti-Lamin B1 (Abcam, ab8982, 1:400), anti-Lamin B1 (Abcam, ab16048, 1:400), anti-H3K9ac (Cell Signaling Technology, 9649, 1:400), anti-H3K27ac (Cell Signaling Technology, 8173, 1:400), anti-H3ac (Abcam, ab47915, 1:400), anti-Tomm20 (Abcam, ab56783, 1:400), anti-Pdha1 (Abcam, ab110334, 1:200), anti-Pcb (Abcam, ab110314, 1:200), anti-Aco2 (Abcam, ab110321, 1:200), anti-Idh3a (Abcam, ab228596, 1:200), anti-Cs (Abcam, ab966000, 1:200), anti-Ogdh (Abcam, ab137773, 1:200), anti-Mdh2 (Abcam, ab110317, 1:200), anti-Sdha (Abcam, ab14715, 1:200), anti-Ssea1 (Santa Cruz, sc-21702, 1:300), anti-Rex1 (Santa Cruz, sc-50668, 1:300), anti-Nanog (Cell Signaling Technology, 4903, 1:300), anti-Mfn2 (Abclonal, A12771, 1:200), anti-Mouse IgG (H + L), alexa fluor 488 (Thermo Fisher Scientific, A11001, 1:400), anti-Mouse IgG (H + L), alexa fluor 568 (Thermo Fisher Scientific, A11004, 1:400), anti-Mouse IgG (H + L), alexa fluor 647 (Thermo Fisher Scientific, A21237, 1:400), anti-Rabbit IgG (H + L), alexa fluor 488 (Thermo Fisher Scientific, A11008, 1:400), anti-Rabbit IgG (H + L), alexa fluor 568 (Thermo Fisher Scientific, A11011, 1:400), anti-Rabbit IgG (H + L), alexa fluor 647(Thermo Fisher Scientific, A21246, 1:400) antibodies were used. The detailed information on antibodies used in this study is shown in Supplementary Table 5.

### Nuclei isolation

Nuclei of cells were isolated using the Nuclei Pure Prep Nuclei Isolation kit (Sigma, NUC201) following the instruction of the manufacturer. The cytoplasmic or nuclei fractions could be separated and nuclei fractions were used for metabolite detection and immunofluorescence. The content of metabolites including pyruvate, citrate, α-KG, and acetyl-CoA was detected immediately after nuclei isolation. For the immunostaining of the TCA cycle enzymes specifically in the nucleus, isolated nuclei were dropped on the coverslips and fixed with 4% PFA immediately after drying and stained according to the above immunofluorescence protocol. The kits used are listed in Supplementary Table 3.

### Cellular pyruvate, acetyl-CoA, citrate, and α-KG quantification

The metabolites of pyruvate, acetyl-CoA, citrate, and α-KG were quantified with the Pyruvate Colorimetric/Fluorometric Assay kit (Biovision, K609), PicoProbe Acetyl-CoA Fluorometric Assay kit (Biovision, K317), Citrate Colorimetric/Fluorometric Assay kit (Biovision, K655) and Alpha-Ketoglutarate Dehydrogenase Activity Assay kit (Biovision, K678), respectively. The whole cells extract, cytoplasm, or nucleus fractions were prepared as described in the nuclei isolation, and the pyruvate, acetyl-CoA, citrate, and α-KG were quantification following the manufacturer’s instruction. For pyruvate and acetyl-CoA measurement, the fluorescence of Excitation/Emission = 535/587 nm was detected. For citrate and α-KG measurement, the absorbance was measured at OD 570 nm for citrate and OD 450 nm for α-KG, respectively.

### Isolation of mitochondria

Mitochondria were isolated using the Qproteome Mitochondria Isolation Kit (Qiagen, 37612) following the manufacturer’s instruction. Nuclei fractions were used for western blot. About 1 × 10^7^ cells were collected and lysed with the lysis buffer at 4 °C for 10 minutes after washing with 0.9% NaCl buffer. The cytosolic proteins as supernatant were collected by centrifuge and the pellet was disrupted by the disruption buffer, and centrifuged (12,000 ×* g*, 4 °C, 15 minutes) to collect nuclei in the pellet. The supernatant was further centrifuged to collect mitochondria in the pellet. The kits are listed in Supplementary Table 3.

### Flow cytometry

Cells were dissociated into single cells using 0.05% trypsin and collected by centrifuging at 300×*g* for 5 minutes. After washing twice with precooled PBS, the cell pellets were collected and resuspended with 100 µl PBS containing 0.1% BSA and 1% FBS. The cells were resuspended and filtered with a 100-well strainer before loading on the FACS (BD Biosciences, LSR Fortessa SORP). Data analysis was performed using FlowJo_V10.

### RNA-seq and data analysis

RNA-seq was performed as our previous report^30^. The total RNA was extracted from cells with TRIzol (Thermo Fisher, 15596018), and libraries were constructed with the VAHTS mRNA-seq v2 Library Prep Kit for Illumina (NR601-01/02, Vazyme) following the manufacturer’s instruction. Sequencing was performed using a Novaseq instrument at HeQin Bio-Technology Co., Ltd (Guangzhou, China). Data analysis was performed by RSEM (v.1.2.22) software and differentially expressed genes were obtained using DESeq2 (v.1.10.1). The value was represented by the modified Fisher’s exact corrected Expression Analysis Systematic Explorer (EASE) score.

### ATAC-see

The assembly of Tn5 transposons was performed as described^29^, and the oligos used are listed in Supplementary Table 4. The cells seeded on coverslips were fixed with 1% formaldehyde (Sigma, 252549) for 10 minutes, and permeabilized with lysis buffer (10 mM Tris, pH 7.4, 10 mM NaCl, 3 mM MgCl_2_, 0.01% Igepal CA-630 (Sigma, I8896) for 10 minutes after rinsing with PBS for three times at room temperature. The assembled transposons were diluted with 25 μl 2×TD buffer (20 mM Tris pH 7.6, 10 mM MgCl_2_, 20% dimethylformamide) and proper volume of ddH_2_O to a total volume of 50 µl with 100 nM transposons. The transposase mixture was added to cells and incubated for 30 minutes at 37 °C. The cells were rinsed three times with washing buffer (1× PBS, 0.01% SDS, 50 mM EDTA, prewarmed at 55 °C for 15 minutes each and mounted after staining with DAPI (1 µg/ml). The imaging was performed on a Zeiss LSM 800 confocal microscope.

### ChIP-qPCR, ChIP-seq, and data analysis

ChIP-qPCR and ChIP-seq were performed following our previous report^30,48^. For the canonical ChIP assay, the cells were cross-linked with 1% formaldehyde (Sigma, 252549) for 10 minutes at room temperature and then quenched with 125 mM glycine (Sigma, G8898). For the two-step cross-linking ChIP assay, cells were cross-linked with 2 mM Disuccinimidyl glutarate (DSG) in PBS supplemented with 1 mM MgCl_2_ for 45 minutes. After washing three times with PBS, cells were cross-linked with 1% formaldehyde and then quenched with 125 mM glycine. The cell lysis, sonication, and immunoprecipitation were performed following the manual of Simple ChIP Enzymatic Chromatin IP Kit (Magnetic Beads) (Cell Signaling Technology, 9003). Data were collected and analyzed as per our previous report^30^. The sequencing reads were mapped to the mouse reference sequence for the mouse genome (mm10) using Bowtie2 (v.2.2.5). Uniquely mapped reads were retained using Samtools (v.1.3.1) and Picard tools MarkDuplicates (v.1.90). Peaks were called using SICER (v.1.1.2) with’W200 G200’ parameters for H3K9ac and H3K27ac modification on histone ChIP-seq data. The signal BigWig files were visualized using computeMatrix, plotHeatmap, and plotProfile modules in DeepTools (v.2.5.4). The BigWig tracks were visualized in the Integrative Genomic Viewer genome (IGV) browser (v.2.4.16). The Gene Ontology analysis was performed using DAVID database (https://david.ncifcrf.gov). The anti-Klf4 (Proteintech, 11880), anti-Sox2 (Cell Signaling Technology, 23064, 1:100), anti-Oct4 (Cell Signaling Technology, 83932,1:100), anti-HA (Cell Signaling Technology, 3724, 1:100), anti-P300 (Cell Signaling Technology, 54062, 1:50), anti-H3K9ac (Cell Signaling Technology, 9649, 1:100), anti-H3K27ac (Cell Signaling Technology, 8173, 1:100), IgG (Cell Signaling Technology, 2729, 1:100) antibodies were used. The primers for ChIP-qPCR used are listed in Supplementary Table 7. The library was constructed following the manual of the kit (Illumina, NQ103). Sequencing was carried out on NovaSeq 6000 by HeQin Bio-Technology Co., Ltd (Guangzhou, China).

### ATAC-seq

About 10^5^ living cells were harvested and sent to HeQin Bio-Technology Co., Ltd for Tn5 treatment and library construction. The ATAC-sequencing and data analysis were performed as our previously report^30,49^. The reads were mapped to the mouse reference sequence mm10 using Bowtie2 (v.2.2.5) with the parameters ‘-X2000 –local’ after filtering with Trimmomatic (v.0.35) and Cutadapt (v.1.13). Uniquely mapped reads were retained using Samtools (v.1.3.1) and Picard tools MarkDuplicates (v.1.90). The peak calling was performed with MACS2 (v.2.1.0) callpeak module, and kept the peaks with *q* value <10^−5^. In each sample, we identified the merged peaks as open or closed by the intersection with sample peaks. Motif enrichment was conducted using the findMotifsGenome.pl module in HOMER (v.4.10.3) with the parameters ‘-len 8,10,12 -size 200’. The signal BigWig files were visualized using computeMatrix, plotHeatmap and plotProfile modules in DeepTools (v.2.5.4). The BigWig tracks were visualized in the Integrative Genomic Viewer genome (IGV) browser (v.2.4.16). The Gene Ontology analysis was performed using DAVID database (https://david.ncifcrf.gov).

### Statistics and reproducibility

Data are presented as mean ± SD. Sample number (*n*) indicates the number of independent biological samples in each experiment except for notes in the figure legend. RNA-seq, ChIP-seq and ATAC-seq were analyzed using a one-way analysis of variance (ANOVA) with Dunnett’s test or a two-way ANOVA with the Sidak correction. Other statistical comparisons were performed using the unpaired two-tailed Student’s *t* tests with GraphPad Prism 8. All experiments were performed at least three times in this study, except where noted in the figure legend. The *p* value <0.05 was considered statistically significant.

### Reporting summary

Further information on research design is available in the Nature Portfolio Reporting Summary linked to this article.

## Supplementary information

Supplementary Information

Reporting Summary

## Data Availability

Data and materials that support the findings are available from the corresponding author on request. The RNA-seq, ChIP-seq, and ATAC-seq data have been deposited in the Genome Sequence Archive (GSA) at the Beijing Institute of Genomics (BIG) Data Center, BIG, Chinese Academy of Sciences. The accession numbers are CRA007453 (RNA-seq), CRA005143 (ChIP-seq data), and CRA005144 (ATAC-seq data) that are publicly accessible at https://bigd.big.ac.cn/gsa. All other relevant data supporting the key findings of this study are available within the article and its Supplementary Information files. Source data are provided with this paper.

## Source data

Source Data
