# Supplementary material for: Nuclear localization of mitochondrial TCA cycle enzymes modulates pluripotency via histone acetylation
Source: Nat Commun. 2022 Dec 2;13:7414. doi: 10.1038/s41467-022-35199-0 (PMC9718843; doi:10.1038/s41467-022-35199-0)
Supplement: Supplementary file 1 — Supplementary Information [file 41467_2022_35199_MOESM1_ESM.pdf]

Nuclear Localization of Mitochondrial TCA Cycle Enzymes Modulates Pluripotency via Histone Acetylation

Wei Li, Qi Long, Hao Wu, Xingguo Liu et al.

Supplementary Information

Supplementary Figure 1-10

Supplementary Table 1-7

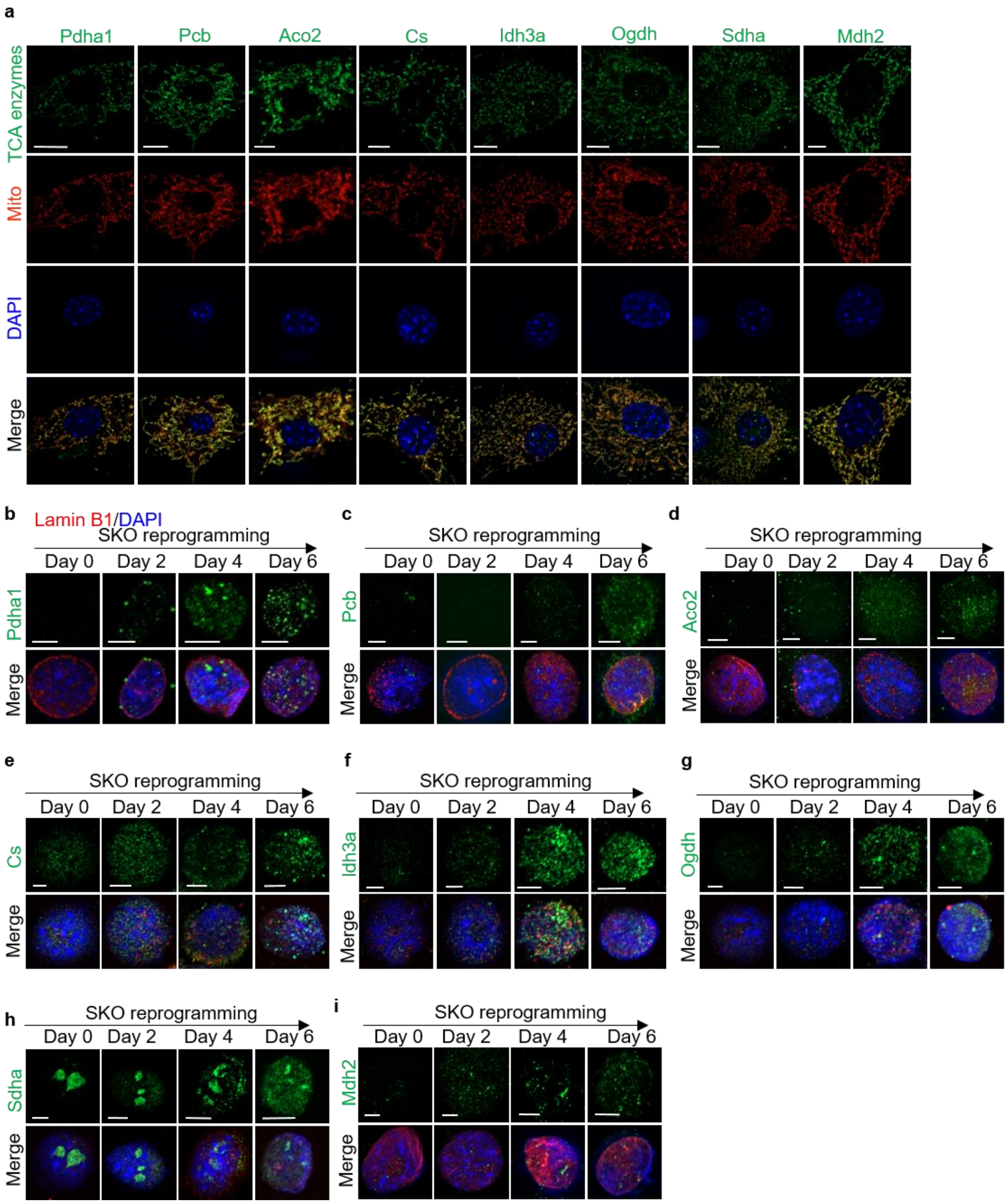

Supplementary Figure 1. TCA Cycle Enzymes in Somatic Cells and Reprogramming Cells. a, Pdha1, Pcb, Aco2, Cs, Idh3a, Ogdh, Sdha and Mdh2 (green) were stained with antibodies in MEFs. The mito-DsRed (Mito) was used as a marker of mitochondria. Scale bars, 5 μm. **b-i,** The translocation of TCA cycle enzymes (purple) at the early stages of somatic cell reprogramming with SKO. The isolated nuclei of cells at day 0 to day 6 during reprogramming were stained with antibodies targeting Pdha1(**b**), Pcb (**c**), Aco2 (**d**), Cs (**e**), Idh3a (**f**), Ogdh (**g**), Sdha (**h**) or Mdh2 (**i**) together with Lamin B1 (red) and DAPI (blue). Scale bars, 5 μm. 3 independent experiments were repeated with similar results and one representative picture was presented.

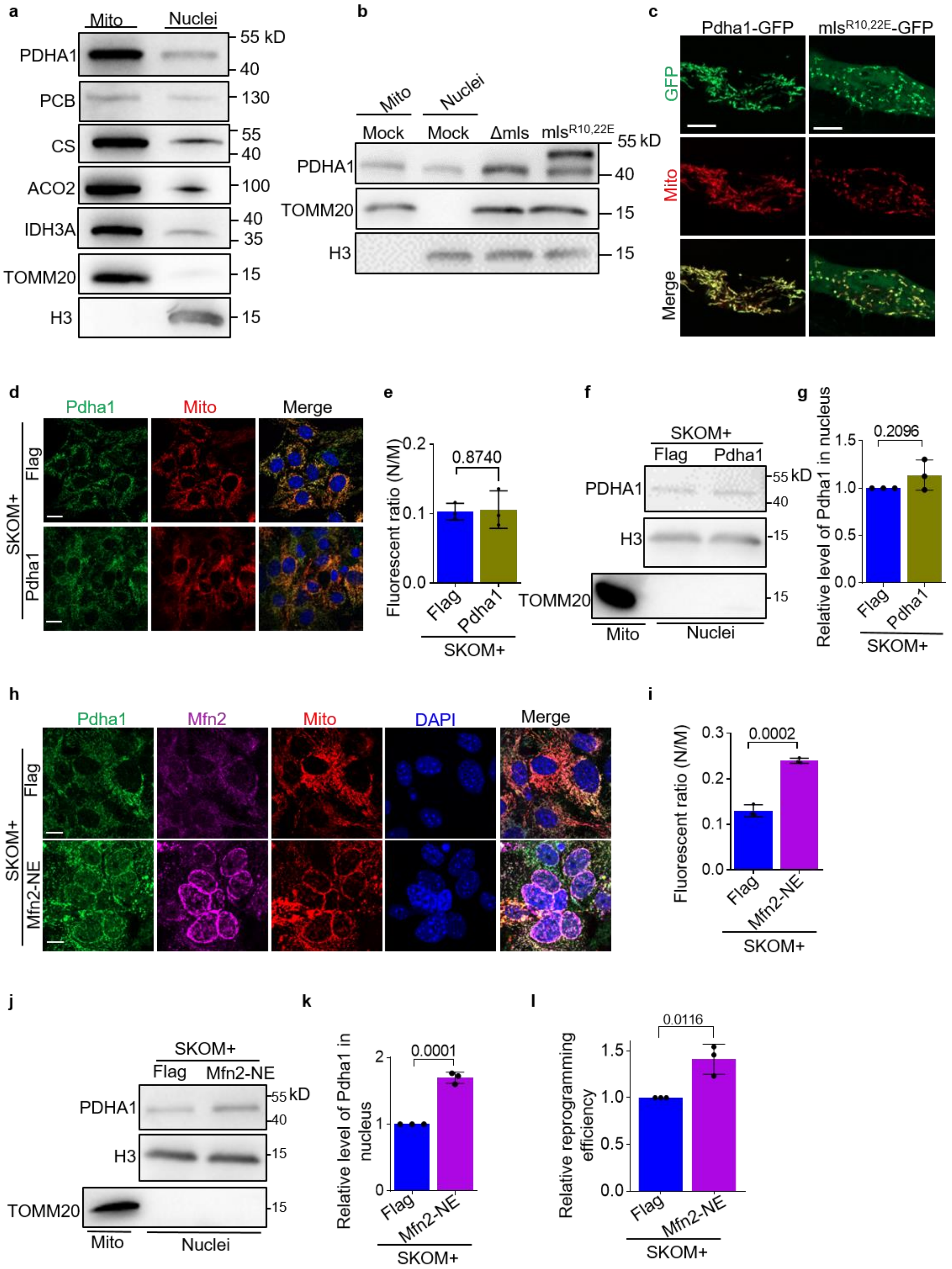

Supplementary Figure 2. The Translocation of TCA Cycle Enzymes to the Nucleus.

a, The western blot analysis of PDHA1, PCB, CS, ACO2 and IDH3A in the fraction of mitochondria or nuclei of ESCs. **b,** The western blot analysis of PDHA1 in the mitochondrial or nuclear fractions of ESCs. The samples transfected with

mitochondrial localization sequence (mls) deletion mutant (Δmls) or mls point mutant (mls^{R10,22E}) of Pdha1 were set as control. **c,** Representative images of Pdha1 or the mls^{R10,22E} mutant. The mito-DsRed (Mito) was used as a marker of mitochondria. Scale bars, 5 μm. **d,** Immunostaining of Pdha1 (green) in MEFs transduced with SKOM plus Pdha1 or Flag on day 4. The mito-DsRed (Mito) was used as a marker of mitochondria. Scale bars, 10 μm. **e,** The count of nuclei/mitochondria fluorescent (N/M) ratio of Pdha1 for (**d**). **f,g,** The western blot of Pdha1 in the nucleus of MEFs transduced with SKOM plus Pdha1 or Flag on day 4 (**f**), as well as the quantification (**g**). **h,** Immunostaining of Pdha1 (green) in MEFs transduced with SKOM plus Mfn2-NE or Flag on day 4. The mito-DsRed (Mito) was used as a marker of mitochondria. Scale bars, 10 μm. **i,** The N/M ratio of Pdha1 in (**h)** were detected. **j,k,** The western blot analysis of Pdha1 in the nucleus of MEFs transduced with SKOM plus Mfn2-NE or Flag on day 4 (**j**), as well as the quantification (**k**). **l,** Relative somatic cell reprogramming efficiency after transduced with Mfn2-NE together with SKOM. The reprogramming efficiency was determined by counting the GFP-positive colonies. The TOMM20 was an indicator of mitochondrial content, while H3 was a loading control (**a**,**b,f,j**). Data are presented as the mean ± S.D and n = 3 independent experiments. At least 30 cells were counted in each experiment (**e**,**i**). A two-tailed unpaired Student's *t*-test was used (**e**,**g,i,k,l**).

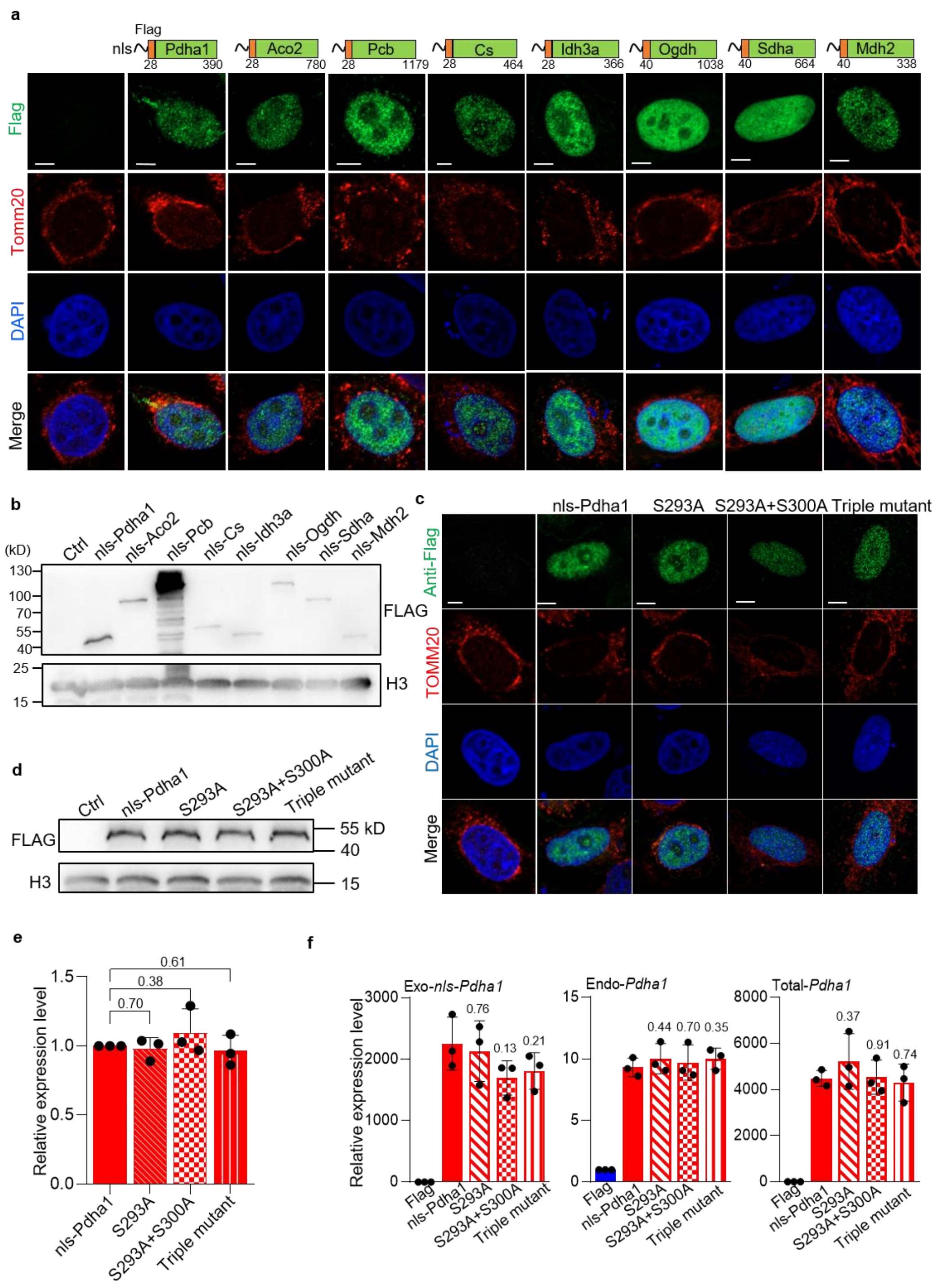

Supplementary Figure 3. The Localization and Overexpression of the Nuclear Localized TCA Cycle Enzymes and Pdha1 Mutants. a, The localization of TCA cycle enzymes as nls-Pdha1, nls-Pcb, nls-Aco2, nls-Cs, nls-Idh3a, nls-Ogdh, nls-Sdha and nls-Mdh2. Schematic diagrams are shown at the top and the immunostaining of

these fusion proteins with anti-Flag antibody (green) are presented below. Scale bars, 5 μm. **b,** The overexpression of nuclear located TCA cycle enzymes were detected by western blot with anti-Flag. **c,** The localization of TCA cycle enzymes nls-Pdha1 and the S->A point mutations in the catalytic subunit of nls-Pdha1 at S232, S293 and S300 including S293A, S293A+S300A or S232A+S293A+S300A (Triple mutant). The immunostaining of these fusion proteins with anti-Flag antibody (green) are detected. Scale bars, 5 μm. **d,e,** ,Western blot (**d**) and quantification (**e**) of MEFs after overexpression nls-Pdha1, or nuclear targeted catalytic mutants of S293A, S293A+S300A or Triple mutant. **f,** The relative expression level of exo-nls-*Pdha1*, endo-*Pdha1* and Total-*Pdha1* were quantified by qPCR after overexpression of nls-Pdha1 or nuclear targeted catalytic mutants of S293A, S293A+S300A or Triple mutant. Cells overexpressing Flag were used as control. Data are presented as mean ± S.D, n = 3 independent experiments (**a**-**f**), and two-tailed unpaired Student's t test were used (**e**-**f**).

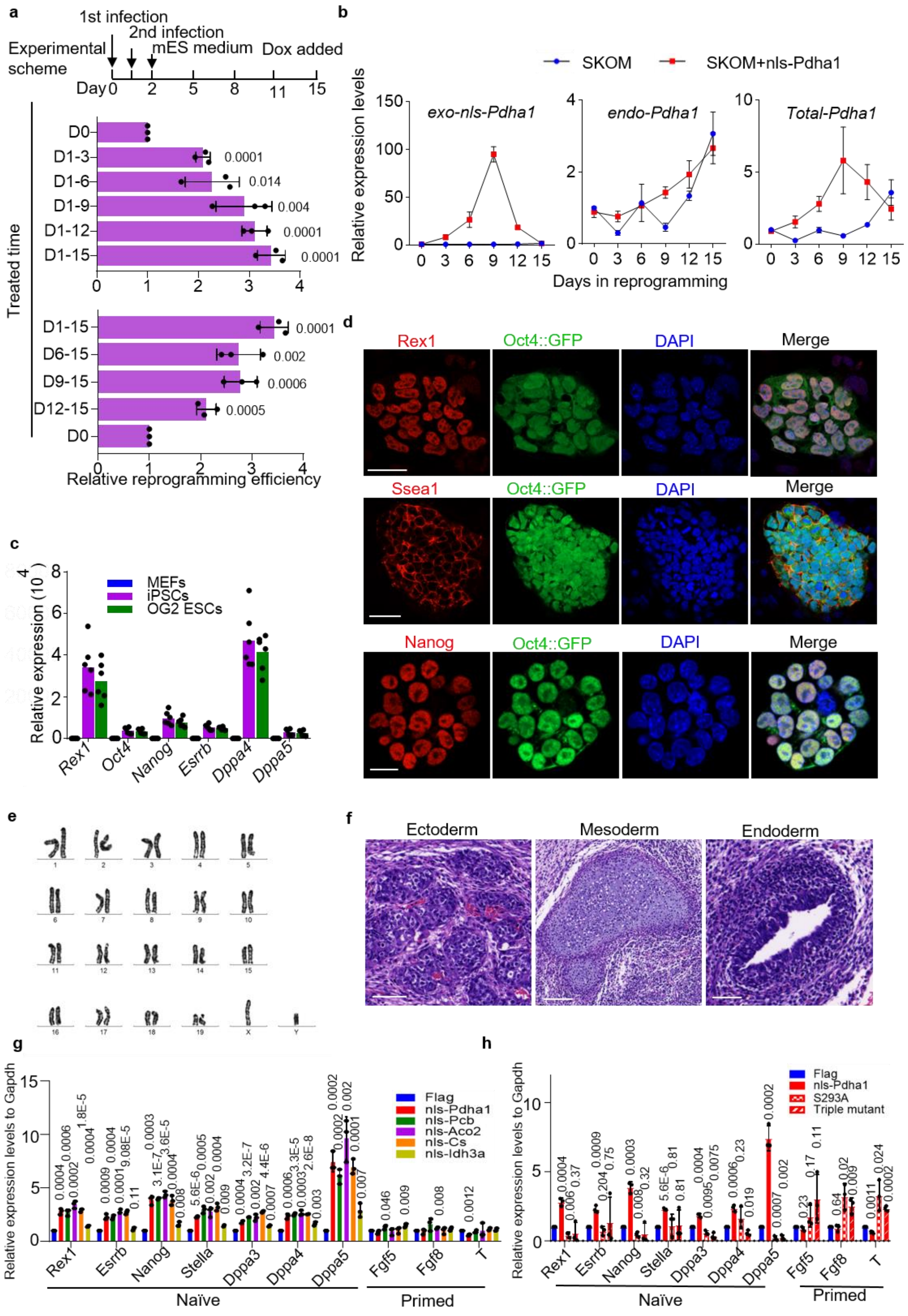

Supplementary Figure 4. Pluripotency Identification of nls-Pdha1-iPSCs a, The reprogramming efficiency with nls-Pdha1 overexpression induced by Dox at different time intervals. DOX was added as indicated and relative reprogramming efficiency were

calculated by compared with DOX-free control. **b,** The relative expression of exo-nls-*Pdha1*, endo-*Pdha1* and Total-*Pdha1* during SKOM-mediated reprogramming with or without nls-Pdha1. **c,** The relative expression of endogenous pluripotency genes (*Rex1*, *Oct4*, *Nanog*, *Esrrb*, *Dppa4* and *Dppa5*). (n = 6 colonies for each group). **d,** Oct4::GFP images and immunofluorescence staining of Rex1, Ssea1 and Nanog (red) for indicating pluripotency in iPSC colonies derived from OG2 MEFs after overexpression nls-Pdha1 (nls-Pdha1-iPSCs). Scale bars, 5 μm. **e,** The karyotype of a representative nls-Pdha1-iPSC colonies. **f,** Three germ layers derived tissues of the teratoma derived from nls-Pdha1-iPSC colonies. Scale bars, 200 μm. **g,** The relative expression level of naïve makers (*Rex1, Esrrb, Nanog, Stella, Dppa3, Dppa4 and Dppa5*) and primed markers (*Fgf5, Fgf8* and *T*) were quantified by qPCR after overexpression of nuclear translocated enzymes including nls-Pdha1, nls-Pcb, nls-Aco2, nls-Cs and nls-Idh3a during PNT. Flag was used as control. **h,** The relative expression level of naïve makers (*Esrrb, Rex1, Oct4, Sox2, Nanog, Stella, Dppa3, Dppa4 and Dppa5*) was quantified by qPCR after overexpression of nls-Pdha1 or nuclear targeted catalytic mutants of Pdha1—S293A or S232A+S293A+S300A (Triple mutant) during PNT. Cells overexpressing Flag were used as control. Data are presented as mean ± S.D, n = 3 independent experiments (**a**, **b**, **d-h**), and two-tailed unpaired Student's t test were used (**a**, **b**, **g, h**).

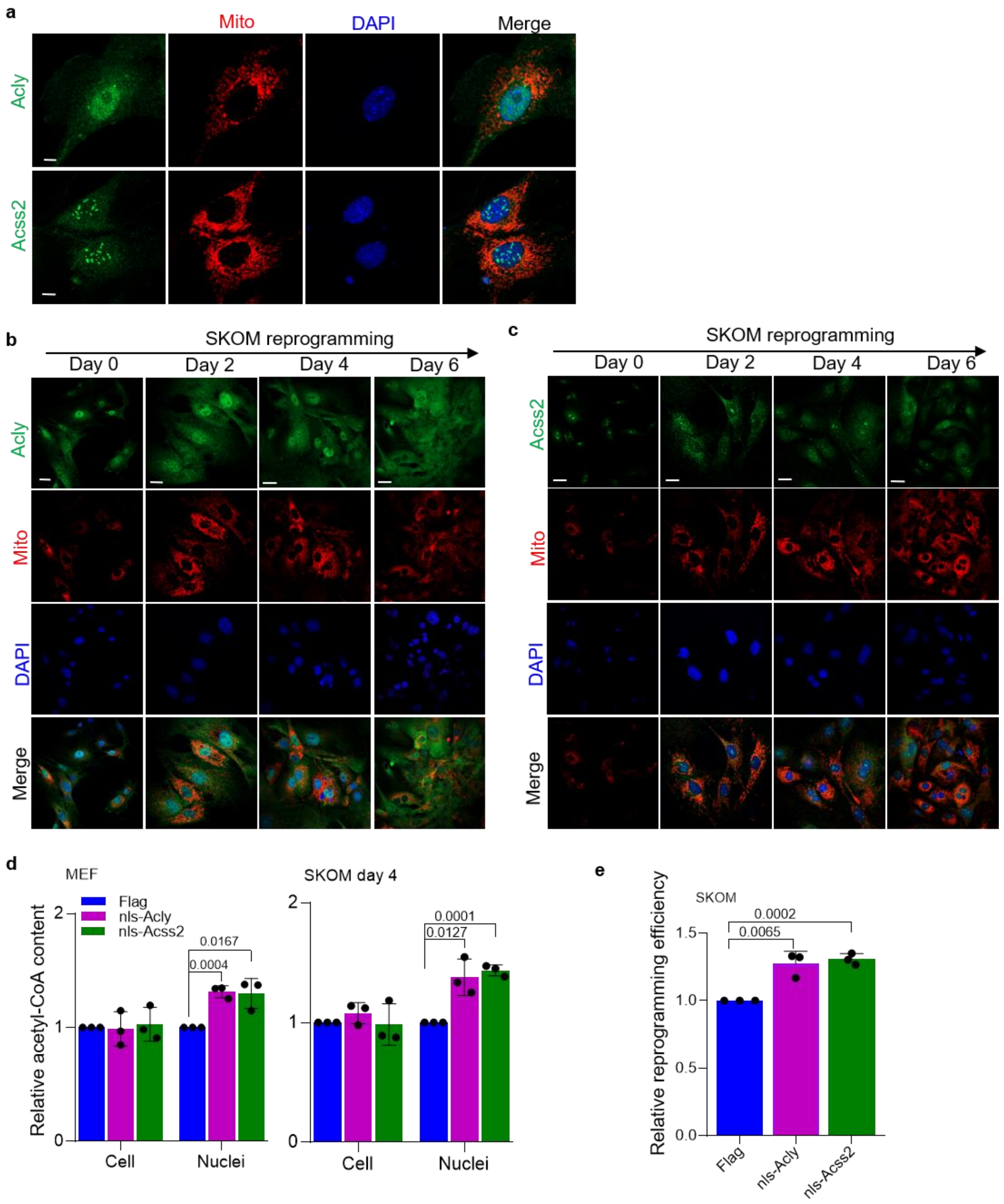

Supplementary Figure 5. The Nuclear Localized Acly and Acss2 Modulates Somatic Cell Reprogramming. a, The localization of Acly and Acss2 in MEFs. Acly and Acss2 were stained with antibodies. The mito-DsRed (Mito) was used as a marker of mitochondria. Scale bars, 5 μm. **b, c,**The localization of Acly (green) (**b**), Acss2 (green) (**c**) during somatic cell reprogramming with SKOM. The mito-DsRed (Mito) was used as a marker of mitochondria. Scale bars, 5 μm. **d,** Relative content of acetyl-

CoA in whole-cell, cytoplasm or nucleus of MEFs in somatic cell reprogramming with SKOM. Cells after overexpression of Flag, nls-Acly or nls-Acss2 were tested. **e,** Relative somatic cell reprogramming efficiency after transducing with nls-Acly or nls-Acss2 together with SKOM. The reprogramming efficiency was determined by counting the GFP-positive colonies. Data are presented as mean ± S.D (**d**-**e**), n = 3 independent experiments (**a**-**e**), and two-tailed unpaired Student's t test were used (**d**-**e**).

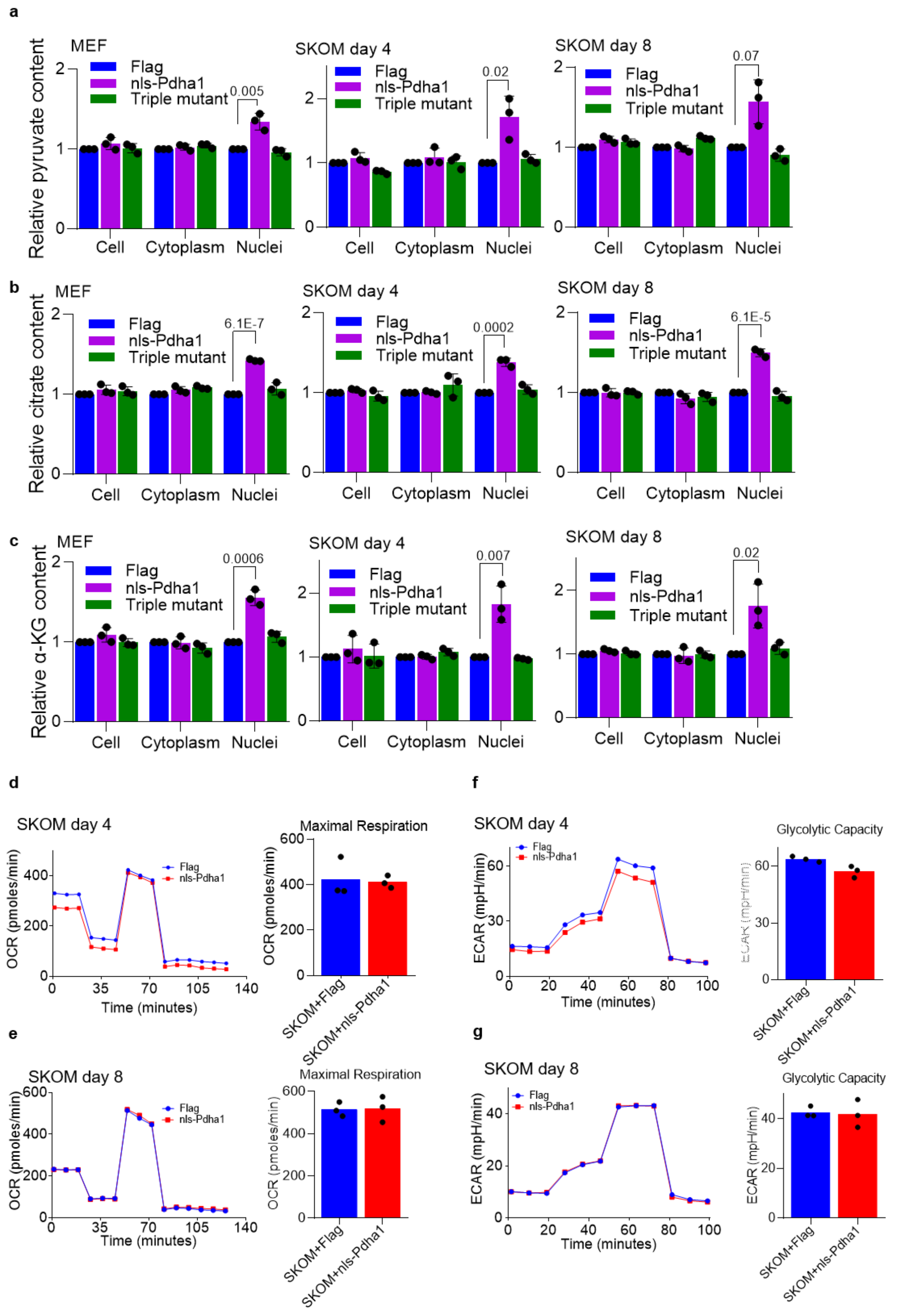

Supplementary Figure 6. Nuclear Pdha1 Promotes the Metabolites of TCA Cycle in the Nucleus during Somatic Cell Reprogramming. a-c, Relative content of pyruvate (**a**), citrate (**b**) or α-KG (**c**) in whole-cell, cytoplasm or nucleus of MEFs

during somatic cell reprogramming with SKOM. Cells after overexpression of Flag, nls-Pdha1 or nls-Pdha1 with triple mutation of its catalytic domain (Triple mutant) were tested. Data are presented as mean ± S.D, n = 3 independent experiments, and two-tailed unpaired Student's t test were used (**a**-**c**). **d,e,** Mitochondrial stress test using Seahorse XF24 on day 4 (**d**) and day 8 (**e**). Oxygen consumption rate (OCR) of mitochondrial maximal respiration was quantified. Reprogramming cell induced by SKOM plus nls-Pdha1 were tested with SKOM plus Flag as control. **f,g,** Glycolysis-stress test on day 4 (**f**) and day 8 (**g**). Extracellular acidification rate (ECAR) was quantified from the glycolysis-stress test. n = 2 independent experiments were repeated with similar results, and one replicate (n = 1, each group) was used for this assay (**d**-**g**).

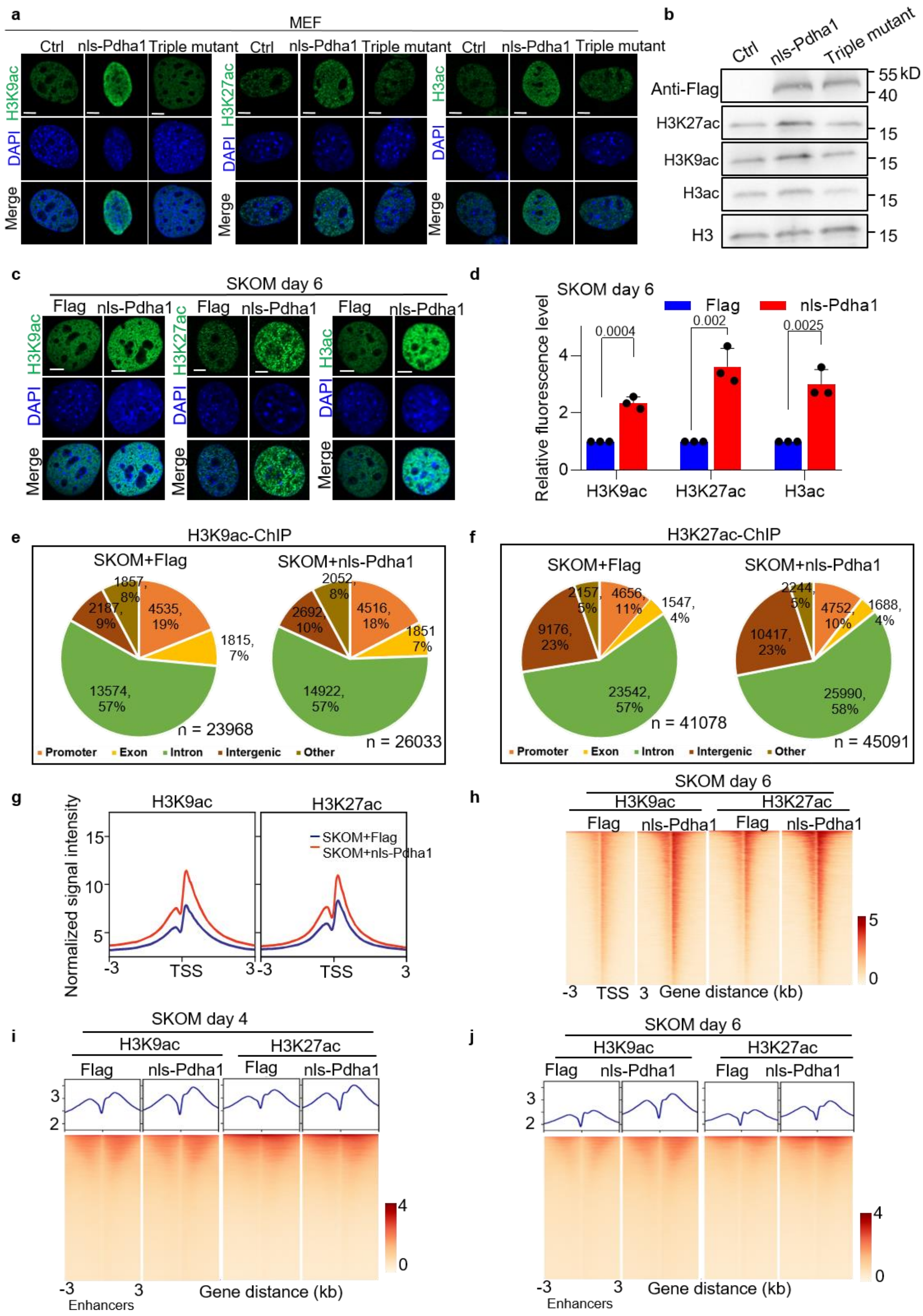

Supplementary Figure 7. Nuclear Pdha1 Promotes Histone Acetylation in Somatic Cell Reprogramming. a, Representative images of H3K9ac, H3K27ac, and H3ac (green) in MEFs plus nls-Pdha1, Triple mutant or Flag (control). Scale bars, 5 μm. **b,** Western blot analysis of H3K9ac, H3K27ac and H3ac in MEFs with nls-Pdha1 or

Triple mutant overexpression. Anti-Flag was targeting nls-Pdha1 and Anti-H3 was used as a loading control. **c,d,** Representative images (**c**) and quantification (**d**) of H3K9ac, H3K27ac, and H3ac (green) at day 6 of cell reprogramming with SKOM plus nls-Pdha1 or Flag (control). Scale bars, 5 μm. **e,f,** Pie charts of H3K9ac (**e**) or H3K27ac (**f**) ChIP-seq data showing the distribution of peaks in MEFs transduced with SKOM with or without nls-Pdha1 overexpression at day 4. **g,h,** Pileup (**g**) and heatmap (**h**) of H3K9ac and H3K27ac ChIP-seq near the TSS in MEFs transduced with SKOM plus nls-Pdha1 or Flag on day 6. n = 2 independent experiments were repeated with similar results (**e-h**), and one replicate (n = 1, each group) was used for this assay (upstream 3 kb and downstream 3 kb of the TSS). **i,j,** Heatmap and pileup of H3K9ac, H3K27ac ChIP-seq near the enhancer in MEFs transduced with SKOM plus nls-Pdha1 or Flag on day 4 (**i**) or day 6 (**j**). n = 2 independent experiments were repeated with similar results, and one replicate (n = 1, each group) was used for this assay (upstream 3 kb and downstream 3 kb of the enhancer) (**i**,**j**). Data are presented as the mean ± S.D (**d**) and n = 3 independent experiments (**a-d**). At least 30 cells were counted in each experiment (**d**). A two-tailed unpaired Student's *t*-test was used (**d**).

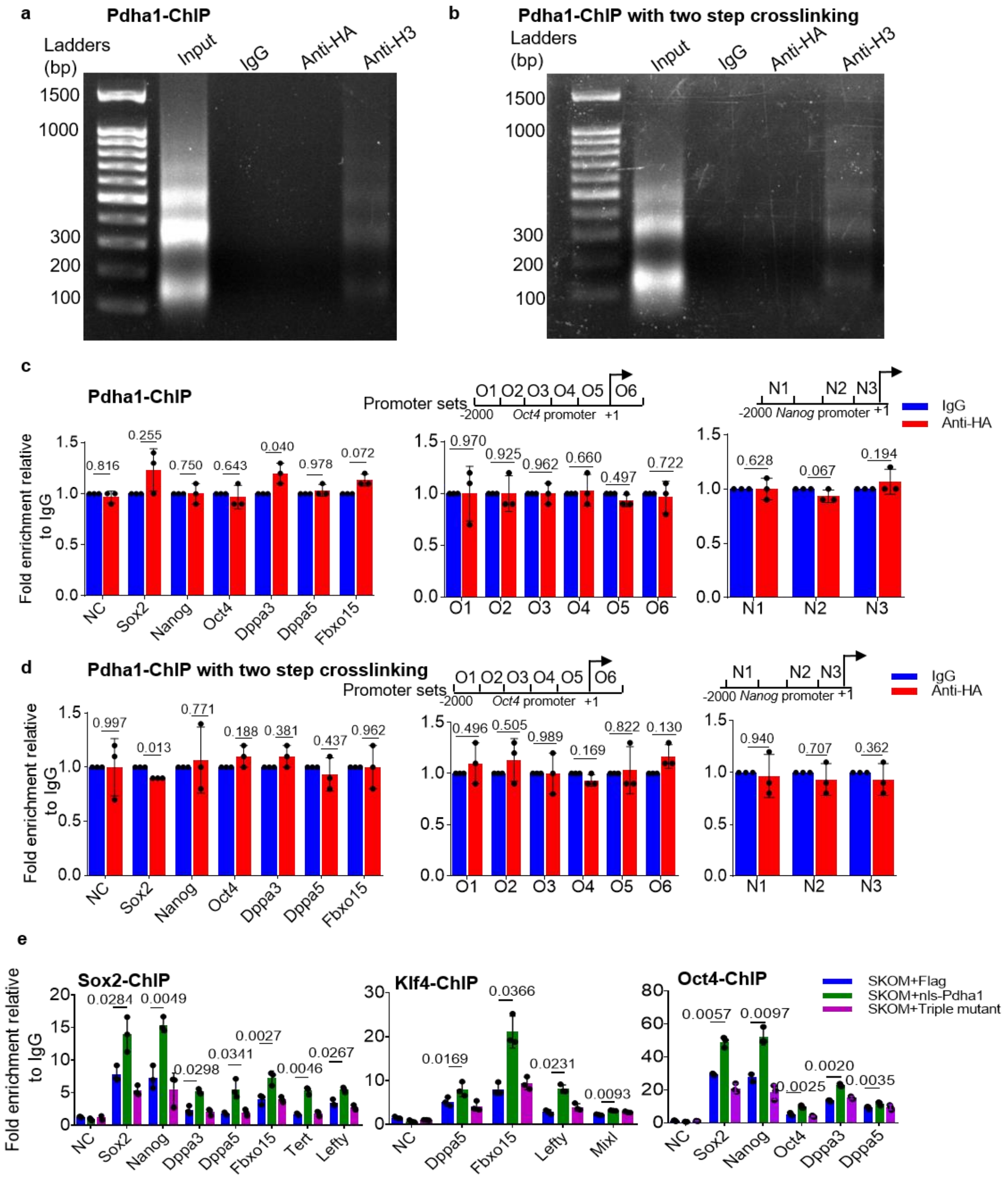

Supplementary Figure 8. The Nuclear Pdha1 Promote Reprogramming with Epigenetic Modulation. a,b, Gel electrophoresis of DNA enriched by ChIP-assay with antibody targeting HA-tag, which was fused with nls-Pdha1 (Input = 2%). A canonical ChIP assay (**a**) or two-step crosslinking ChIP assay (**b**) were performed. Anti-H3 was set as a positive control. About 4×10^6 cells after transduced with SKOM plus nls-Pdha1 on day 4 were collected**. c,** ChIP-qPCR analysis of nls-Pdha1 enrichment on promoter regions of pluripotency genes (*Sox2*, *Nanog*, *Oct4*, *Dppa3*, *Dppa5*, *Fbxo15*). The promoter of *Oct4* (O1-O6) and *Nanog* (N1-3) were detailed. **d**, Two-step cross-linking

ChIP assay about the nls-Pdha1 enrichment on promoter regions of pluripotency genes (*Sox2*, *Nanog*, *Oct4*, *Dppa3*, *Dppa5*, *Fbxo15*). The promoter of *Oct4* (O1-O6) and *Nanog* (N1-3) were detailed. **e,** ChIP-qPCR assay in detecting the binding of Sox2, Klf4 and Oct4 to their respective targets in MEFs infected with SKOM plus Flag, nls-Pdha1 or Triple mutant. Data are presented as the mean ± S.D (**c**-**e**) and n = 3 independent experiments (**a**-**e**). A two-tailed unpaired Student's *t*-test was used (**c**-**e**).

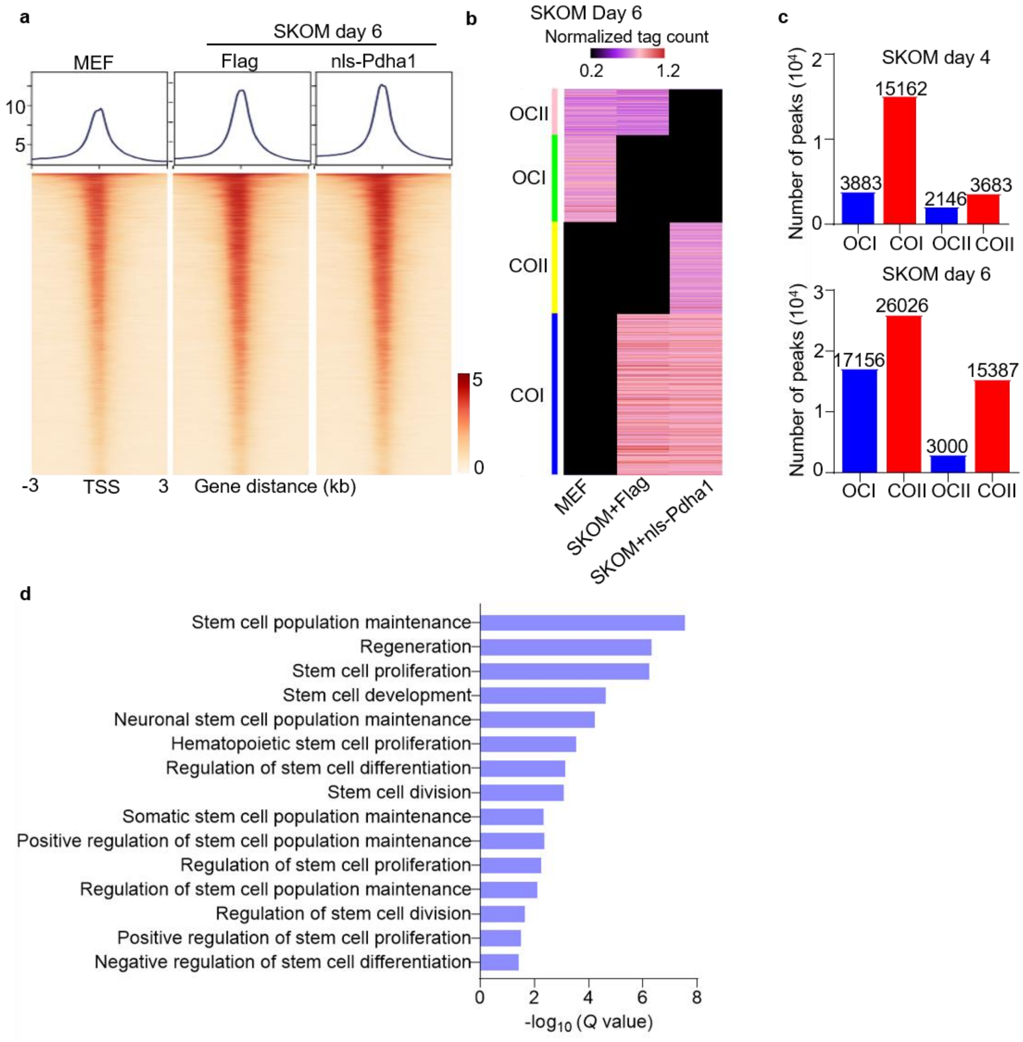

Supplementary Figure 9. Nuclear Pdha1 Loosens Chromatin. a, Heatmap and pileup of ATAC-seq signal near the TSS in MEFs and MEFs transduced with SKOM plus nls-Pdha1 or Flag on day 6. Three replicates (n = 3, each group) were used for ATAC assay. (Upstream 3 kb and downstream 3 kb of the TSS). **b,** Heatmap of ATAC–seq OC/CO loci on day 6 during reprogramming: closed in Flag but open in SKOM+Flag and SKOM+nls-Pdha1 (COI), closed in Flag and SKOM+Flag but open in SKOM+nls-Pdha1 (COII), open in Flag but closed in SKOM+Flag and SKOM+nls-Pdha1 (OCI), open in Flag and SKOM+Flag but closed in SKOM+nls-Pdha1 (OCII). Units are in normalized sequence tag counts. Three replicates (n = 3, each group) were used for the ATAC assay. **c,** The number of peaks of each type by ATAC-seq on day 4 (up panel) and day 6 (down panel) (COI/OCI/COII/OCII). **d,** GO analysis for COII peaks of ATAC-seq defined in **Fig. 6d** on day 4 during reprogramming. 3 independent

experiments were repeated with similar results, and one replicate (n = 1, each group) was used for this analysis (**b-e**).

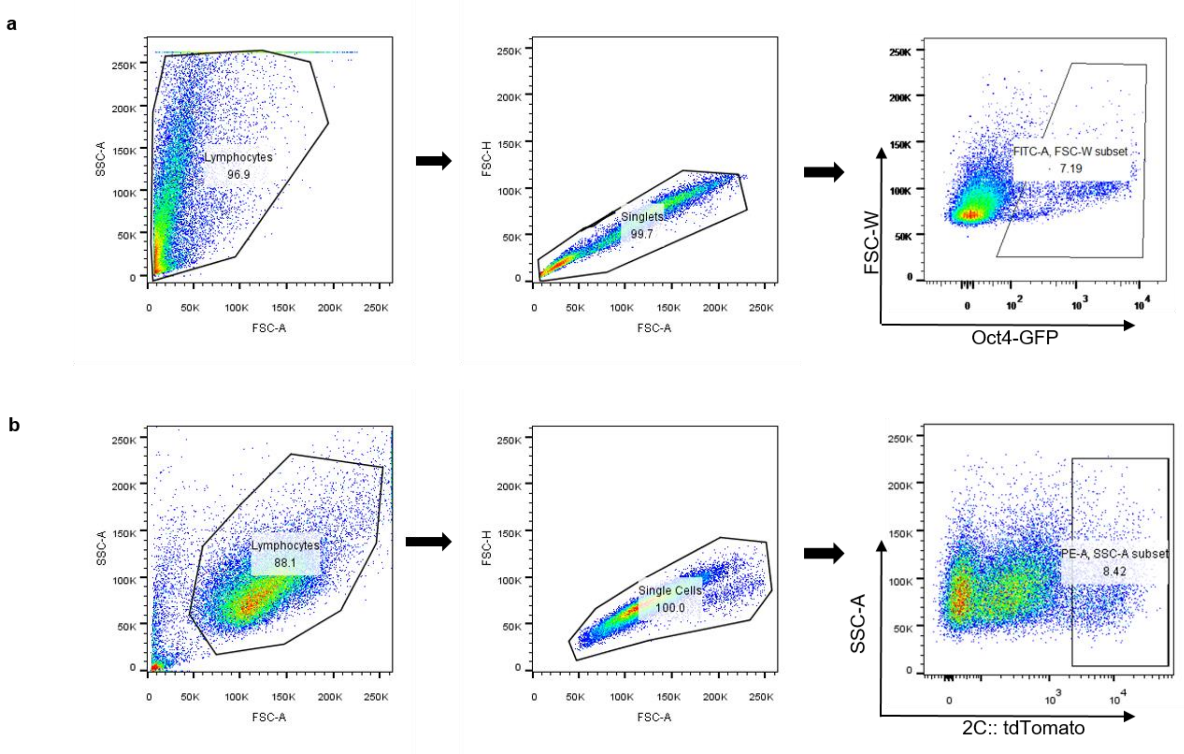

Supplementary Figure 10. FACS gating strategy **a.** FACS gating strategy for flow cytometry analysis of GFP positive naïve stem cell colonies generated from EpiSCs with nls-Pdha1, nls-Pdha1-mutant (S293A, Triple mutant), nls-Pcb, nls-Aco2, nls-Cs or nls-Idh3a overexpression. in Fig. 3g, i. **b.** FACS gating strategy for flow cytometry analysis (gated on 2C:: tdTomato cells) showing the relative proportion of 2C:: tdTomato-positive totipotency stem cells generated from OG2 ESCs with overexpression of nls-Pdha1 or nls-Pdha1-mutant (S293A, Triple mutant) in Fig. 3k.

Supplementary Table 1. The plasmids used in this paper.

Plasmids	Source	Catalog
pMXs-Sox2	Addgene	13367
pMXs-Klf4	Addgene	13370
pMXs-Oct3/4	Addgene	13366
pMXs-cMyc	Addgene	13375
pMXs-Pdha1	This paper	N/A
pMXs-nls-3xFlag-Pdha1	This paper	N/A
pMXs-nls-3xFlag-Pcb	This paper	N/A
pMXs-nls-3xFlag-Aco2	This paper	N/A
pMXs-nls-3xFlag-Cs	This paper	N/A
pMXs-nls-3xFlag-Idh3a	This paper	N/A
pMXs-nls-3xFlag-Ogdh	This paper	N/A
pMXs-nls-3xFlag-Sdha	This paper	N/A
pMXs-nls-3xFlag-Mdh2	This paper	N/A
pMXs-nls-3xFlag-Pdha1-S293A	This paper	N/A
pMXs-nls-3xFlag-Pdha1-(S239A+S300A)	This paper	N/A
pMXs-nls-3xFlag-Pdha1-(S232A+S239A+S300A)	This paper	N/A
pMXs- nls-HA-Pdha1	This paper	N/A
pMXs-Δmls-Pdha1	This paper	N/A
pMXs-mls^{R10,22E} -Pdha1	This paper	N/A
pMXs-Pdha1-GFP	This paper	N/A
pMXs-mls^{R10,22E} -Pdha1-GFP	This paper	N/A
pMXs-Mfn2-NE	This paper	N/A
pMXs-nls-3xFlag-Acly	This paper	N/A
pMXs-nls-3xFlag-Acss2	This paper	N/A
pW-nls-Pdha1	This paper	N/A

pMXs-mitoDsRed	This paper	N/A
pRLenti-mitoDsred	This paper	N/A
pRLenti-3xFlag-puro	This paper	N/A
pRLenti-nls-3xFlag-Pdha1-puro	This paper	N/A
pRLenti-nls-3xFlag-Pcb-puro	This paper	N/A
pRLenti-nls-3xFlag-Aco2-puro	This paper	N/A
pRLenti-nls-3xFlag-Cs-puro	This paper	N/A
pRLenti-nls-3xFlag-Idh3a-puro	This paper	N/A
pRLenti-nls-3xFlag-Pdha1- S293A -puro	This paper	N/A
pRLenti-nls-3xFlag-Pdha1-(S232A+S239A+S300A)-puro	This paper	N/A
psPAX2	Addgene	12260
pMD2.G	Addgene	12259

Supplementary Table 2. The reagent used in this paper.

Reagent	Source	Catalog
DMEM-Dulbecco's Modified Eagle Medium, High Glucose	Gibco	11995500BT
FBS	Gibco	10099141
GlutaMAX	Gibco	35050079
Non-Essential Amino Acids Solution (NEAA)	Gibco	11140076
Sodium Pyruvate Solution	Gibco	11360070
N2	Gibco	17502048
B27	Gibco	17504044
β-Mercaptoethoethanol	Gibco	21985-023
Penicillin-Streptomycin Solution	Hyclone	SV30010
CHIR99021	Selleck	S1263
PD0325901	Selleck	S1036
XAV-939	Selleck	284028-89-3
bFGF	R&D Systems	3139-FB-025/CF
polyethyleneimine	PolyScience	23966-1
Activin A	Novoprotein	C687
Trypsin-EDTA (0.25%), phenol red	Gibco	25200114
Trypsin-EDTA (0.05%), phenol red	Gibco	25300054
Formaldehyde	Sigma	252549
Igepal CA-630	Sigma	I8896
Glycine	Sigma	G8898
RIPA	Beyotime	P0013B
Disuccinimidyl glutarate (DSG)	HARVEYBIO	
PMSF	Beyotime	ST506
TRIzol	Thermo Fisher	15596018
Triton X-100	Sigma	T8787

DAPI	Sigma	D9542
Giemsa stain	Coolaber	Sl7010
Fluorescence mounting medium	DAKO	S3023
protease inhibitor cocktail	Roche	04693116001
ECL solution	Millipore	WBKLS0500

Supplementary Table 3. The kit used in this paper.

Kit	Source	Catalog
SYBR Green QPCR kit	TaKaRa	RR820A
Reverse Transcription kit	TaKaRa	RR047A
RNA Purification kit	EZBioscience	B0004DP
Seahorse XF Cell Mito Stress Test Kit	Agilent Technologies	103015-100
Seahorse XF Glycolysis Stress Test Kit	Agilent Technologies	103020-100
H&E Staining Kit	Abcam	Ab245880
Nuclei Pure Prep Nuclei Isolation kit	Sigma	NUC201
SimpleChIP Enzymatic Chromatin IP Kit (Magnetic Beads)	CST	9003
Nuclear and Cytoplasmic Protein Extraction kit	Beyotime	P0028
PicoProbe AcetylCoA Fluorometric Assay kit	Biovision	K317
Pyruvate Colorimetric/Fluorometric Assay kit	Biovision	K609
Citrate Colorimetric/Fluorometric Assay kit	Biovision	K655
Alpha-Ketoglutarate Dehydrogenase Activity Assay kit	Biovision	K678
Qproteome Mitochondria Isolation Kit	Qiagen	37612

Supplementary Table 4. The oligos used in this paper.

Tn5ME-A-ATTO590	TCGTCGGCAGCGTCAGATGTGTATAAGAGACAG
Tn5ME-B-ATTO590	GTCTCGTGGGCTCGGAGATGTGTATAAGAGACAG
Tn5MErev	[phos]CTGTCTCTTATACACATCT

Supplementary Table 5. The antibodies used in this paper.

Antibodies	Company	Catalog
Anti-Lamin B1	Abcam	ab8982
Anti-Lamin B1	Abcam	ab16048
Anti-Histone H3 antibody	Abcam	ab1791
Anti-Histone H3K27ac antibody	CST	8173
Anti-Histone H3K9ac antibody	CST	9649
Anti-Histone H3 (acetyl K9 + K14 + K18 + K23 + K27)	Abcam	ab47915
Anti-IgG	CST	2729
Anti-Histone H3K36me3 antibody	Abcam	ab194677
Anti-Histone H3K27me3 antibody	Abcam	ab272165
Anti-Histone H3K9me3 antibody	Abcam	ab8898
Anti-Tomm20 antibody	Abcam	ab56783
Anti-Pdha1	Abcam	ab110334
Anti-PCB[3H2AD9]	Abcam	ab110314
Anti-PCB antibody-C-terminal	Abcam	ab229267
Anti-Aconitase 2[6F12BD9]	Abcam	ab110321
Anti-Idh3a antibody	Abcam	ab228596
Anti-Citrate synthetase	Abcam	ab96600
Anti-OGDH- C-terminal	Abcam	ab137773
Anti-MDH_2[2F5AF8]	Abcam	ab110317
Anti-Sdha	Abcam	ab14715
Anti-FLAG	SIGMA	F1804
Anti-SSEA1 antibody	Santa Cruz	sc-21702
Anti-Rex1 antibody	Santa Cruz	sc-50668
Anti-Nanog	CST	4903S
Anti-P300	CST	54062

Anti-Sox2	CST	23064
Anti-Oct4	CST	83932
Anti-Klf4	Proteintech	11880
Anti-HA	CST	3724
Anti-Mfn2	Abclonal	A12771
Goat anti-Mouse IgG (H+L), Alexa Fluor 488	Invitrogen	A11001
Goat anti-Mouse IgG (H+L), Alexa Fluor 568	Invitrogen	A11004
Goat anti-Rabbit IgG (H+L), Alexa Fluor 488	Invitrogen	A11008
Goat anti-Rabbit IgG (H+L), Alexa Fluor 568	Invitrogen	A11011
Goat anti-Mouse IgG (H+L), Alexa Fluor 647	Invitrogen	A21237
Goat anti-Rabbit IgG (H+L), Alexa Fluor 647	Invitrogen	A21246
HRP-conjugated goat anti-mouse antibody	Kangchen	KC-MM-035
HRP-conjugated goat anti-rabbit antibody	Kangchen	KC-RB-035

Supplementary Table 6. Primers for Q-PCR in this paper.

Gapdh	Forward	AACTTTGGCATTGTGGAAGGGCTCA
	Reverse	TTGGCAGCACCAGTGGATGCAGGGA
Actin	Forward	GTGACGTTGACATCCGTAAAGA
	Reverse	GCCGGACTCATCGTACTCC
Sox2	Forward	TTCGAGGAAAGGGTTCTTGCTG
	Reverse	TCCTTCCTTGTTTGTAACGGTCCT
Oct4	Forward	TGTTCCCGTCACTGCTCTGG
	Reverse	TTGCCTTGGCTCACAGCATC
Rex1	Forward	GGCTGCGAGAAGAGCTTTATTCA
	Reverse	AGCATTTCTTCCCGGCCTTT
Stella	Forward	GCCGCACAGCAGATGTGAA
	Reverse	AAATCTGGATCGTTGTGCATCCT
Esrrb	Forward	GCACCTGGGCTCTAGTTGC
	Reverse	TACAGTCCTCGTAGCTCTTGC
Nanog	Forward	AAATCCCTTCCCTCGCCATC
	Reverse	GCGTTCCCAGAATTCGATGC
Dppa3	Forward	TGTGGAGAACAAGAGTGA
	Reverse	CTCAATCCGAACAAGTCTT
Dppa4	*Forward*	GCAGATGTCTAGTCAACCAAGC
	Reverse	TCCTGGCGTCTCAGTGTCT
Dppa5	*Forward*	GCGATAGCCCAAAGAAGT
	Reverse	ACAGAGATTGAAGCAGACAT
Fgf5	Forward	CCTTGCGACCCAGGAGCTTA
	Reverse	CCGTCTGTGGTTTCTGTTGAGG
Fgf8	*Forward*	TCGCGAAGCTCATTGTGGA
	Reverse	GCCGTTGCTCTTGGCAATTAG

T	*Forward*	TTGAACTTTCCTCCATGTGCTGA
	Reverse	TCCCAAGAGCCTGCCACTTT
Exo-*nls-Pdha1*	Forward	AAGCGGAAGGTCGACTACAA
	Reverse	GCCCTCTTCTAGCCGATGAA
Endo-*Pdha1*	*Forward*	GTCGGTTCCCAGTCCATCAG
	Reverse	GCACATGACATTTCTGTTGCG
Total-*Pdha1*	*Forward*	AGGCTGGCATAAACCCTACG
	Reverse	GCCGTTGCCTCCATAGAAGT

Supplementary Table 7. Primers for ChIP-QPCR in this paper.

Gapdh-ChIP	Forward	CCTTCATTGACCTCAACTACA
	Reverse	TAGACTCCACGACATACTCA
Sox2-ChIP	Forward	TTTATTCAGTTCCCAGTCCAA
	Reverse	TTATTCCTATGTGTGAGCAAGA
Oct4-ChIP	Forward	ATACTTGAACTGTGGTGGAG
	Reverse	CTATCATGCACCTTTGTTAT
Nanog-ChIP	Forward	AGGTGGGAAGTATCTATGG
	Reverse	CGGCTATTCTATTCAGTGG
Setbp1-ChIP	Forward	ATGTCCCAAAGCCAACAA
	Reverse	TCATCCCACCTGACCACC
Col1a1-ChIP	Forward	GGGCCTCTAACAAATCTC
	Reverse	CAGGAAGCCTGTGACCAG
Col5a1-ChIP	Forward	ACTCCTGTCTCCCTCTGC
	Reverse	CGCCTTACTGCTGTCCCT
NC-ChIP	Forward	AGCATGTGTTCTTCTTACCA
	Reverse	GTTAGTTCATATTATTGTTCCACCTA
Dppa3-ChIP	Forward	AATCAAGTAGGGAAAGAAAGGA
	Reverse	AAGGCACAGTTACACTCTTAT
Dppa5-ChIP	Forward	GCGATAGCCCAAAGAAGT
	Reverse	ACAGAGATTGAAGCAGACAT
Mixl-ChIP	Forward	GAATAATCGCTTCCGCTGAC
	Reverse	AGAGGGGGTTCTGTCCAAGT
Tert-ChIP	Forward	ACTTTGGTTGCCCAATGC
	Reverse	AAGGAAAGGTCGGCAGGT
Chd1-ChIP	Forward	CCATGTTAAAATGTCATTTA
	Reverse	TGGAGTTACAAAGGACTTTA

Lefty-ChIP	Forward	GTCCAGACAGGCTTTTGTGT
	Reverse	GTCTGCGGAGGAATGGTA
Fbxo15-ChIP	Forward	GCCCTTAGTTCCCAGATG
	Reverse	CTCACCTTACAAGTCCTCAA
O1-ChIP	Forward	CTCTCGTCCTAGCCCTTCCT
	Reverse	CCTCCACTCTGTCATGCTCA
O2-ChIP	Forward	CTGACCCTAGCCAACAGCTC
	Reverse	TGCTCCTACACCATGCTCTG
O3-ChIP	Forward	CTTAGTGTCTTTCCGCCAGC
	Reverse	TCCCCTCACACAAGACTTCC
O4-ChIP	Forward	GCACTTCTCTGGGGTCTCTG
	Reverse	TGAACCCAGTATTTCAGCCC
O5-ChIP	Forward	CTGTAAGGACAGGCCGAGAG
	Reverse	CAGGAGGCCTTCATTTTCAA
O6-ChIP	Forward	CACGAGTGGAAAGCAACTCA
	Reverse	TTGGTTCCACCTTCTCCAAC
N1-ChIP	Forward	ATCGCCTTGAGCCGTTGG
	Reverse	CGAGGGAAGGGATTTCTG
N2-ChIP	Forward	ATGGTGGCTGTGGTGGC
	Reverse	GGTTGGTGGTGTTTGTTTGA
N3-ChIP	Forward	GGCAGTGGAAGAAGGGAA
	Reverse	AGCCACCATACTACTACTGTCTC
