# Supplementary material for: Nuclear localization of mitochondrial TCA cycle enzymes modulates pluripotency via histone acetylation
Source: Nat Commun. 2022 Dec 2;13:7414. doi: 10.1038/s41467-022-35199-0 (PMC9718843; doi:10.1038/s41467-022-35199-0)
Supplement: Supplementary file 2 — Reporting Summary [file 41467_2022_35199_MOESM2_ESM.pdf]

nature research

Corresponding author(s): Xingguo Liu

Last updated by author(s): 22/11/22

Reporting Summary

Nature Research wishes to improve the reproducibility of the work that we publish. This form provides structure for consistency and transparency in reporting. For further information on Nature Research policies, see our Editorial Policies and the Editorial Policy Checklist.

Statistics

For all statistical analyses, confirm that the following items are present in the figure legend, table legend, main text, or Methods section.

n/a	Confirmed	
☐	☒	The exact sample size (*n*) for each experimental group/condition, given as a discrete number and unit of measurement
☐	☒	A statement on whether measurements were taken from distinct samples or whether the same sample was measured repeatedly
☐	☒	The statistical test(s) used AND whether they are one- or two-sided<br>*Only common tests should be described solely by name; describe more complex techniques in the Methods section.*
☒	☐	A description of all covariates tested
☒	☐	A description of any assumptions or corrections, such as tests of normality and adjustment for multiple comparisons
☐	☒	A full description of the statistical parameters including central tendency (e.g. means) or other basic estimates (e.g. regression coefficient) AND variation (e.g. standard deviation) or associated estimates of uncertainty (e.g. confidence intervals)
☐	☒	For null hypothesis testing, the test statistic (e.g. *F*, *t*, *r*) with confidence intervals, effect sizes, degrees of freedom and *P* value noted<br>*Give P values as exact values whenever suitable.*
☒	☐	For Bayesian analysis, information on the choice of priors and Markov chain Monte Carlo settings
☒	☐	For hierarchical and complex designs, identification of the appropriate level for tests and full reporting of outcomes
☒	☐	Estimates of effect sizes (e.g. Cohen's *d*, Pearson's *r*), indicating how they were calculated

Our web collection on statistics for biologists contains articles on many of the points above.

Software and code

Policy information about availability of computer code

Data collection	RNA-sequencmg reads, ChIP-sequencing reads and ATAC-sequencing reads were generated at lllumina NovaSeq 6000 platform (HeQin Bio-Technology Co., Ltd , Guangzhou, China).<br>Extracellular Metabolic Flux Analysis were performed by an XFe24 Analyzer system (Seahorse Bioscience).<br>Quantitative PCR was performed in a CFX96 Real-Time System (Bio-Rad).<br>The Flow Cytometry analysis were performed by LSR Fortessa SORP, BD Biosciences.<br>Confocal images were collected with inverted confocal microscope (LSM800, LSM880).<br>Western blot signals were detected using a Mini Chemi910 Chemiluminescent/ Fluorescent Imaging and Analysis System (SageCreation�Beijing, China).
Data analysis	Bowtie2 software (version 2.2.5), Samtools (1.3.1), MarkDuplicates (1.90), DeepTools (2.5.4)and the Integrative Genom1cs Viewer (IGV) (2.4.16) were used to analyze ChIP-seq data. The Gene Ontology analysis was performed using DAVID database (https:// david.ncifcrf.gov).<br>Bowtie2 (2.2.5), Samtools (1.3.1), MarkDuplicates (1.90),MACS2 (2.1.0),DeepTools(2.5.4), HOMER (v.4.10.3), IGV (2.4.16) were used to analyze ATAC-seq data. The Gene Ontology analysis was performed using DAVID database (https:// david.ncifcrf.gov).<br>ImageJ 1.52a were used to analyze western blot bands density.<br>FlowJo_V10 were used to analyze the Flow Cytometry data.<br>GraphPad Prism 8 were used to analyze statistical data.

For manuscripts utilizing custom algorithms or software that are central to the research but not yet described in published literature, software must be made available to editors and reviewers. We strongly encourage code deposition in a community repository (e.g. GitHub). See the Nature Research guidelines for submitting code & software for further information.

Data

Policy information about availability of data

All manuscripts must include a data availability statement. This statement should provide the following information, where applicable:

- Accession codes, unique identifiers, or web links for publicly available datasets
- A list of figures that have associated raw data
- A description of any restrictions on data availability

Data and materials that support the findings are available from the corresponding author on request. The RNA-seq, ChIP-seq and ATAC-seq data have been deposited in the Genome Sequence Archive (GSA) at the Beijing Institute of Genomics (BIG) Data Center, BIG, Chinese Academy of Sciences. The accession numbers are CRA007453 (RNA-seq), CRA005143 (ChIP-seq data) and CRA005144 (ATAC-seq data) that are publicly accessible at https://bigd.big.ac.cn/gsa. All other relevant data supporting the key findings of this study are available within the article and its Supplementary Information files. Source data are provided with this paper.

Field-specific reporting

Please select the one below that is the best fit for your research. If you are not sure, read the appropriate sections before making your selection.

☒ Life sciences ☐ Behavioural & social sciences ☐ Ecological, evolutionary & environmental sciences

For a reference copy of the document with all sections, see nature.com/documents/nr-reporting-summary-flat.pdf

Life sciences study design

All studies must disclose on these points even when the disclosure is negative.

Sample size	The sample size was estimated from the preliminary experiments or from our previously published studies. No statistical method was applied to predetermine sample size. The RT-qPCR, FACS, Western blot and Immunofluorescence were conducted with at least three independent experiments except otherwise stated. RNA-seq, ChIP-seq and ATAC-seq were conducted with two or three independent samples. Animal or embryo experiments have been performed at least three times independently. Please Refer to figure legends and methods for details. All replications were successful.
Data exclusions	No data were excluded for analyses.
Replication	All the sequencing includes at least two independent biological replicates; all of other experiments includes at least three biological replicates. We have added detailed description of replication in each experiment in Figure legends or Methods. The data exclusion criteria is described above.
Randomization	For the pluripotency identification, the colonies of iPSCs were picked up at random. No randomization methods were utilized for other experiments as all samples are randomly collected from the population, sample collections were performed with controls, all replications were successful and no data were excluded.
Blinding	Experiments execution, data collection and result analysis were usually carried out by the same person, there to be no blinding was used. Plus, this study does not include animals or human research participants. Furthermore, data analysis was carried out mainly bioinformatically without need for investigator blinding

Reporting for specific materials, systems and methods

We require information from authors about some types of materials, experimental systems and methods used in many studies. Here, indicate whether each material, system or method listed is relevant to your study. If you are not sure if a list item applies to your research, read the appropriate section before selecting a response.

Materials & experimental systems

n/a	Involved in the study	
☐	☒	Antibodies
☐	☒	Eukaryotic cell lines
☒	☐	Palaeontology and archaeology
☐	☒	Animals and other organisms
☒	☐	Human research participants
☒	☐	Clinical data
☒	☐	Dual use research of concern

Methods

n/a	Involved in the study	
☐	☒	ChIP-seq
☐	☒	Flow cytometry
☒	☐	MRI-based neuroimaging

Antibodies

Antibodies used	Lamin B1 (Abcam, ab8982, 1:400 dilution for Immunofluorescence),

Antibodies used

Lamin B1 (Abcam, ab16048, 1:400 dilution for Immunofluorescence),
H3 (Abcam, ab1791, 1:5000 dilution for WB),
H3K27ac (Cell Signaling Technology, #8173, 1:3000 dilution for WB, 1:400 dilution for Immunofluorescence, 1:100 for ChIP-seq),
H3K9ac (Cell Signaling Technology, #9649, 1:3000 dilution for WB, 1:400 dilution for Immunofluorescence, 1:100 for ChIP-seq),
P300 (Cell Signaling Technology,#54062, 1:50 dilution for ChIP),
Klf4 (Proteintech, 11880,1:100 dilution for ChIP),
Sox2 (Cell Signaling Technology, 23064,1:100 dilution for ChIP),
Oct4 (Cell Signaling Technology, 83932,1:100 dilution for ChIP),
HA (Cell Signaling Technology, 3724,1:100 dilution for ChIP),
IgG (Cell Signaling Technology, 2729,1:100 dilution for ChIP),
H3ac (Abcam, ab47915, 1:3000 dilution for WB, 1:400 dilution for Immunofluorescence),
H3K36me3 (Abcam, ab194677, 1:3000 dilution for WB),
H3K27me3 (Abcam, ab272165, 1:3000 dilution for WB),
H3K9me3 (Abcam, ab8898, 1:3000 dilution for WB),
Tomm20 (Abcam, ab56783, 1:3000 dilution for WB, 1:400 dilution for Immunofluorescence),
Pdha1 (Abcam, ab110334, 1:1000 dilution for WB, 1:200 dilution for Immunofluorescence),
Pcb (Abcam, ab110314, 1:200 dilution for Immunofluorescence),
Pcb (Abcam, ab229267, 1:1000 dilution for WB),
Aco2 (Abcam, ab110321, 1:1000 dilution for WB, 1:200 dilution for Immunofluorescence),
Idh3a (Abcam, ab228596, 1:1000 dilution for WB, 1:200 dilution for Immunofluorescence),
Cs (Abcam, ab966000, 1:1000 dilution for WB, 1:200 dilution for Immunofluorescence),
Ogdh (Abcam, ab137773, 1:200 dilution for Immunofluorescence),
Mdh2 (Abcam, ab110317, 1:200 dilution for Immunofluorescence),
Sdha (Abcam, ab14715, 1:200 dilution for Immunofluorescence),
anti-Flag (Sigma, F1804, 1:2000 dilution for WB),
Ssea1(Santa Cruz, sc-21702, 1:300 dilution for Immunofluorescence),
Rex1(Santa Cruz, sc-50668, 1:300 dilution for Immunofluorescence),
Nanog (Cell Signaling Technology, 4903,1:300 dilution for Immunofluorescence),
Mfn2(abclonal,A12771 1:200 dilution for Immunofluorescence),
anti-Mouse IgG (H+L), alexa fluor 488 (Thermo Fisher Scientific, A11001, 1:400 dilution for Immunofluorescence),
anti-Mouse IgG (H+L), alexa fluor 568 (Thermo Fisher Scientific, A11004, 1:400 dilution for Immunofluorescence),
anti-Mouse IgG (H+L), alexa fluor 647 (Thermo Fisher Scientific, A21237, 1:400 dilution for Immunofluorescence),
anti-Rabbit IgG (H+L), alexa fluor 488 (Thermo Fisher Scientific, A11008, 1:400 dilution for Immunofluorescence),
anti-Rabbit IgG (H+L), alexa fluor 568 (Thermo Fisher Scientific, A11011, 1:400 dilution for Immunofluorescence),
anti-Rabbit IgG (H+L), alexa fluor 647(Thermo Fisher Scientific, A21246, 1:400 dilution for Immunofluorescence),
HRP-conjugated goat anti-mouse antibody (Kangchen, KC-MM-035, 1:4000 dilution for WB),
HRP-conjugated goat anti-rabbit antibody (Kangchen, KC-RB-035, 1:4000 dilution for WB),

Validation

Antibody validation for Western blot analyses involved confirmation that the band corresponded to the reported molecular mass following gel migration.
Antibody validation for Immunofluorescence analyses involved confirmation that corresponded to the reported protein location.
Antibodies against H3K9ac, H3K27ac, p300, Sox2, Oct4, HA-Tag, IgG for ChIP analyses involved confirmation by the below web site of Cell Signaling Technology.
Antibodies against Klf4 for ChIP analyses involved confirmation by the bewlow web site of Proteintech.
https://www.cellsignal.com/products/primary-antibodies/acetyl-histone-h3-lys9-c5b11-rabbit-mab/9649?site-search-type=Products&N=4294956287&Ntt=h3k9ac&fromPage=plp
https://www.cellsignal.com/products/primary-antibodies/acetyl-histone-h3-lys27-d5e4-xp-rabbit-mab/8173?site-search-type=Products&N=4294956287&Ntt=h3k27ac&fromPage=plp
https://www.cellsignal.com/products/primary-antibodies/p300-d2x6n-rabbit-mab/54062?site-search-type=Products&N=4294956287&Ntt=p300&fromPage=plp
https://www.cellsignal.com/products/primary-antibodies/sox2-d9b8n-rabbit-mab/23064?site-search-type=Products&N=4294956287&Ntt=sox2&fromPage=plp
https://www.cellsignal.com/products/primary-antibodies/oct-4a-d6c8t-rabbit-mab-mouse-specific/83932?site-search-type=Products&N=4294956287&Ntt=oct4&fromPage=plp
https://www.cellsignal.com/products/primary-antibodies/ha-tag-c29f4-rabbit-mab/3724?site-search-type=Products&N=4294956287&Ntt=ha&fromPage=plp
https://www.cellsignal.com/products/primary-antibodies/normal-rabbit-igg/2729
https://www.ptglab.com/Products/KLF4-Antibody-11880-1-AP.htm

Eukaryotic cell lines

Policy information about cell lines

Cell line source(s)

Plat-E (ATCC; RRID: CVCL_0063), HEK293T (ATCC, catalog no. CRL-3216), and Hela (ATCC, catalog no. CCL-2) were purchased from ATCC. ICR-MEF, OG2-MEF, EpiSCs and ESCs were were isolated and cultured in our institute.

Authentication

Plat-E and HEK293T Cell lines were derived from authenticated stock from ATCC.
ICR-MEF and OG2-MEF cell are primary cells isolated from mice.
Hela cell line was authenticated in 2019 through STR DNA profiling analysis.

Mycoplasma contamination

Cells were all monthly tested and devoid of mycoplasma contamination.

Commonly misidentified lines (See ICLAC register) | No commonly misidentified cells were used.

Animals and other organisms

Policy information about studies involving animals; ARRIVE guidelines recommended for reporting animal research

Laboratory animals	The 6-8 week-old female BALB/c-Nude mice were used in the study.All mice have free access to food and water and were housed in a pathogen-free environment with a 12:12 dark/light cycle, controlled temperature (23±2°C) and humidity (60±10%).
Wild animals	The study did not involve wild animals
Field-collected samples	The study did not involve samples collected from field.
Ethics oversight	All animal experiments were performed according to guidelines the Care and Use of Laboratory Animals of the National Instate of Health, and the protocol was the experimental were performed according to the protocol N2022106 approved by the Institutional Animal Care and Use Committee (IACUC) of Guangzhou Institutes of Biomedicine and Health, CAS.

Note that full information on the approval of the study protocol must also be provided in the manuscript.

ChIP-seq

Data deposition

☒ Confirm that both raw and final processed data have been deposited in a public database such as GEO.

☒ Confirm that you have deposited or provided access to graph files (e.g. BED files) for the called peaks.

Data access links<br>*May remain private before publication.*	https://ngdc.cncb.ac.cn/gsa/browse/CRA005143
Files in database submission	SKOM+Flag-D3Input replicate1<br>SKOM+Flag-D3H3K9ac replicate1<br>SKOM+Flag-D3H3K27ac replicate1<br>SKOM+nls-Pdha1-D3Input replicate1<br>SKOM+nls-Pdha1-D3H3K9ac replicate1<br>SKOM+nls-Pdha1-D3H3K27ac replicate1<br>SKOM+Flag-D5Input replicate1<br>SKOM+Flag-D5H3K9ac replicate1<br>SKOM+Flag-D5H3K27ac replicate1<br>SKOM+nls-Pdha1-D5Input replicate1<br>SKOM+nls-Pdha1-D5H3K9ac replicate1<br>SKOM+nls-Pdha1-D5H3K27ac replicate1<br>SKOM+Flag-D3Input replicate2<br>SKOM+Flag-D3H3K9ac replicate2<br>SKOM+Flag-D3H3K27ac replicate2<br>SKOM+nls-Pdha1-D3Input replicate2<br>SKOM+nls-Pdha1-D3H3K9ac replicate2<br>SKOM+nls-Pdha1-D3H3K27ac replicate2<br>SKOM+Flag-D5Input replicate2<br>SKOM+Flag-D5H3K9ac replicate2<br>SKOM+Flag-D5H3K27ac replicate2<br>SKOM+nls-Pdha1-D5Input replicate2<br>SKOM+nls-Pdha1-D5H3K9ac replicate2<br>SKOM+nls-Pdha1-D5H3K27ac replicate2
Genome browser session<br>(e.g. UCSC)	Not applicable.

Methodology

Replicates	There are two biological replicates.
Sequencing depth	Reads are paired end, 150 bp reads, with a estimated fragment length of 100-500 bp in size.
Antibodies	The following antibodies were used for ChIP-seq experiments:<br>ChIP-seq antibodies: anti-IgG (Cell Signaling Technology, #2729, 1:100 dilution for ChIP-seq),<br>H3K9ac (Cell Signaling Technology, #9649, 1:100 for ChIP-seq),<br>H3K27Ac (Cell Signaling Technology, #8173, 1:100 for ChIP-seq),

Peak calling parameters	Peaks were called using SICER (1.1.2) with "W200 G200" parameters for H3K9ac H3K27ac modification.
Data quality	No H3K9ac and H3K27ac peaks are at an estimated FDR level of >5% and all reported peaks are >5 fold, as reported by DFilter.
Software	The sequencing reads were filtered by Trimmomatic (0.35), and then mapped to mouse reference sequence for mouse genome (mm10) using Bowtie2 (2.2.5).<br>Uniquely mapped reads were retained using Samtools (v.1.3.1) and Picard tools MarkDuplicates (v.1.90).<br>ChIPpeakAnno (3.16.1) was used for identifying nearby genes from the peaks obtained from MACS2 (2.1.0).<br>The signal BigWig files were visualized using in DeepTools (2.5.4).<br>The BigWig tracks were visualized in the Integrative Genomic Viewer genome (IGV) browser (v.2.4.16).

Flow Cytometry

Plots

Confirm that:

☒ The axis labels state the marker and fluorochrome used (e.g. CD4-FITC).

☒ The axis scales are clearly visible. Include numbers along axes only for bottom left plot of group (a 'group' is an analysis of identical markers).

☒ All plots are contour plots with outliers or pseudocolor plots.

☒ A numerical value for number of cells or percentage (with statistics) is provided.

Methodology

Sample preparation	Cells were dissociated into single cells using 0.05% trypsin and collected by centrifuging at 1000 rpm for 5 minutes. After washing twice with precooled PBS⍰the cell pellets were collected and resuspended with 100 μl PBS containing 0.1% BSA and 1% FBS. The cells were resuspended and filtered with a 100-well strainer.
Instrument	A LSR Fortessa SORP machine was used for data collection.
Software	Flowjo (10.4)
Cell population abundance	A minimum of 10000 cells were counted per sample analyzed
Gating strategy	Every flow cytometry analysis was initiated as follows:FSC-A/SSC-A gates to gate the lymphocyte population; FSC-A/FSC-H to select sigle cells.; followed by the gating as described in the Figures.

☒ Tick this box to confirm that a figure exemplifying the gating strategy is provided in the Supplementary Information.
